# From Populations to Programming Variables: Post‐Activation Performance Enhancement Following Isometric Contractions on Muscular Strength and Power—A Systematic Review and Meta‐Analysis

**DOI:** 10.1002/ejsc.70183

**Published:** 2026-05-19

**Authors:** Zhijian He, Zhipeng Xue, Lijun Qiang, Zepeng Lu, Qiushi Wang, Ruinan Zhang, Yilin Xu, Ji Tu, Dapeng Bao, Junhong Zhou

**Affiliations:** ^1^ Department of Sports Teaching and Research Lanzhou University Lanzhou China; ^2^ School of Strength and Conditioning Training Beijing Sport University Beijing China; ^3^ China Institute of Sport and Health Science Beijing Sport University Beijing China; ^4^ Ningxia Vocational College of Sports Ningxia China; ^5^ Sports Coaching College Beijing Sport University Beijing China; ^6^ China Basketball College Beijing Sport University Beijing China; ^7^ Institute of Basic Medical Sciences Chinese Academy of Medical Sciences School of Basic Medicine Peking Union Medical College Beijing China; ^8^ Medical Examination Center Peking University Third Hospital Beijing China; ^9^ Hebrew SeniorLife Hinda and Arthur Marcus Institute for Aging Research Harvard Medical School Boston Massachusetts USA

**Keywords:** holding isometric muscle action, isometric squat, pushing isometric muscle action, rest interval, vertical jump

## Abstract

Isometric contraction (ISO) has emerged as a potentially effective conditioning activity (CA) for inducing post‐activation performance enhancement (PAPE). However, findings regarding its effects on subsequent muscular strength and power remain inconsistent, and the participant‐ and protocol‐related characteristics associated with more favorable responses remain unclear. This systematic‐review and meta‐analysis aimed to evaluate the acute effects of ISO on subsequent muscular strength and power, compared ISO with control (CON) and dynamic isotonic contraction (DYN) conditions, and explore characteristics associated with more favorable PAPE. Twenty‐five studies involving 782 participants were included. A three‐level meta‐analysis was used to account for dependent effect sizes, complemented by moderator analyses and meta‐regression. For vertical jump height, ISO showed a significantly greater effect than CON (ES = 0.349, *p* < 0.01), whereas no significant difference was detected between ISO and DYN; a significant pre–post improvement was observed within the ISO group (ES = 0.180, *p* < 0.01). No significant within‐ISO effect was observed for power output, and no significant effect was observed for muscular strength in either the within‐ISO or ISO versus CON comparison. Moderator and meta‐regression analyses suggested that some participant‐ and protocol‐related conditions were associated with larger subgroup‐specific effects on the vertical jump height, particularly 8–12 s contraction duration, 6–10 volume units, and 4–8 min of rest, although between‐level differences were generally inconsistent. ISO appears effective for eliciting PAPE in vertical jump height, whereas current evidence for muscular strength and power remains limited.

**Protocol Registration:**

The original protocol for the review was prospectively registered on PROSPERO (CRD42024608192)

## Highlights

- Isometric contractions, performed after a general warm‐up, appear effective for eliciting PAPE in vertical jump height, and it may potentially serve as a practical alternative to dynamic isotonic contractions.
- Moderator analyses suggested that subgroup‐specific effects tended to be greater in males, in participants with ≥ 2 years of strength training experience, and in protocols characterized by certain joint angles, intensity, and volume profiles.
- Larger average effects were generally observed with 8–12 s contractions, 6–10 volume units, and 4–8 min of rest. Both HIMA and PIMA were associated with significant subgroup‐specific effects.

## Graphical abstract

This systematic review and three‐level meta‐analysis examined the acute effects of isometric contractions as conditioning activities for post‐activation performance enhancement. The findings suggest that isometric contractions may produce a small but significant improvement in vertical jump height, whereas the evidence for muscular strength and power output remains limited or inconclusive. Practical application should consider the balance between potentiation and fatigue, as well as contraction duration, volume, rest interval, joint angle, isometric contraction modality, and participant characteristics.

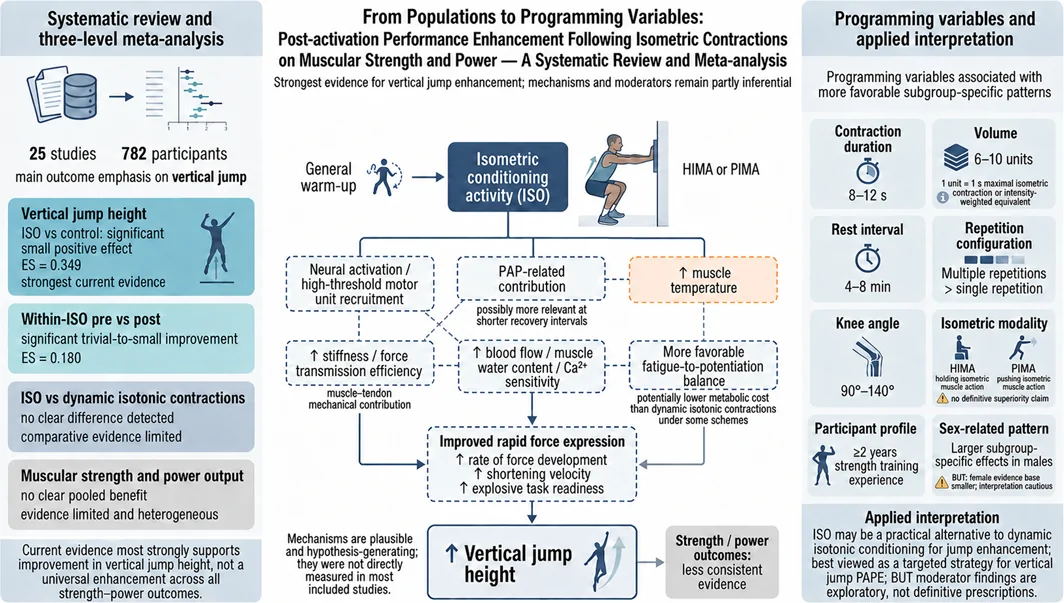

## 1 Introduction

Post‐activation performance enhancement (PAPE), rather than post‐activation potentiation (PAP, increased electrically evoked force such as the twitch response after prior voluntary contraction), is defined as the acute improvement in voluntary muscular performance (e.g., force or power output) following a conditioning activity (CA) (Blazevich and Babault 2019; Stone et al. 2008; DeRenne and Journal 2010; Ebben WPJJoss, 2002; Mihalik et al. 2008). In training settings, a range of CA protocols have been developed to induce PAPE by attempting to optimize the net balance between fatigue and potentiation (Rassier et al. 2000; Sale 2002; Sale DJBjosm 2004). Among these, high‐intensity dynamic isotonic contractions (≥ 85% one repetition maximum, [1RM]) are the most widely studied and have been shown to enhance subsequent explosive performance (Blazevich and Babault 2019; Xu et al. 2024; Seitz and Haff 2016; Tillin and Bishop 2009). However, their applications in real‐world training settings may be constrained due to concerns about exercise setup, protocol timing, and contextual feasibility (Boullosa 2021). As highlighted in recent reviews and survey‐based evidence, the translation of PAPE protocols from controlled research settings to field‐based practice remains challenging (Boullosa 2021; Sungur et al. 2026). Therefore, recent studies have increasingly explored alternative conditioning activity protocols to identify more practical and efficient strategies for facilitating PAPE (Kasicki et al. 2024; Koźlenia and Domaradzki 2023a; Sun et al. 2024; Koźlenia and Domaradzki 2023b). Isometric contraction (ISO) has emerged as a potentially effective strategy for inducing PAPE. ISO involves the production of force against a constant resistance without an observable change in muscle length (Oranchuk, Storey, Nelson, and Cronin 2019a; Schaefer and Bittmann 2017; Folland et al. 2005). Current evidence indicates that ISO, particularly when performed with high intensity and ballistic intent, may be associated with favorable neuromuscular activation characteristics (Oranchuk, Storey, Nelson, and Cronin 2019a; Linnamo et al. 2003) and mechanical properties related to stiffness, which may in turn benefit force transmission and rapid force expression (Kubo et al. 1985. 2001). Furthermore, under specific loading schemes, ISO may impose a lower metabolic demand and/or provide a more favorable fatigue‐to‐potentiation balance than dynamic isotonic contractions (Varela‐Olalla et al. 2025; Vedsted et al. 2003; Elder et al. 2006).

Several studies have shown that isometric contraction can induce PAPE in various strength‐ and power‐related outcomes, such as peak force, countermovement jump height, and sprint performance (Rixon et al. 2007; Ino et al. 2024; Downey et al. 2022; Tsoukos et al. 2016; D PIPER et al. 2020; French et al. 2003). However, some studies also showed insignificant PAPE effect of ISO on muscular strength and power (Herring et al. 2021; Spieszny et al. 2022; Koźlenia and JJFiP 2023; Vargas‐Molina et al. 2021). For example, one study showed a significant improvement in vertical jump performance after a 2‐min rest interval following a 7‐s isometric back squat at 85% 1RM in individuals with at least 1 year of strength training experience (Downey et al. 2022). In contrast, Herring et al. reported that maximal isometric mid‐thigh pulls (IMTP) produced a negative effect on CMJ and 30 m sprint performance after a rest interval of 4–7 min and 8–10 min, respectively, in participants with certain strength level and training experience (Herring et al. 2021). This inconsistency may be partly explained by variations in training intensity and rest interval, as current evidence suggests that both factors may influence the net balance between fatigue and potentiation responses (Xu et al. 2024; Seitz and Haff 2016; Tillin and Bishop 2009). In addition, participants with different strength training experiences and strength levels may exhibit distinct responses, potentially due to differences in the proportion and cross‐sectional area of type II muscle fibers, as well as fatigue‐resistance characteristics (Blazevich and Babault 2019; Seitz and Haff 2016). Recent literature has distinguished between holding isometric muscle action (HIMA) and pushing isometric muscle action (PIMA), as they may differ in their neuromuscular and cardiovascular/metabolic outputs (Oranchuk et al. 2024). Taken together, these findings suggest that the effects of isometric contraction on subsequent performance are likely moderated by participant characteristics (Seitz and Haff 2016; Koźlenia and JJFiP 2023; Seitz et al. 2014), protocol design, task selection, and recovery timing (Seitz and Haff 2016; Tillin and Bishop 2009; Tsoukos et al. 2016; French et al. 2003; Lum and Barbosa 2019; Batista et al. 2011; Trimble and Harp 1998; Vandervoort et al. 1983; T. J. Allen et al. 1985. 2018; Guex et al. 2013). To the best of our knowledge, no systematic review or meta‐analysis has specifically synthesized the effects of ISO used as conditioning activities across strength, power, and jump outcomes using multilevel models and protocol moderators.

Therefore, the aim of this systematic review and meta‐analysis was to comprehensively summarize the PAPE of isometric contraction on muscular strength and power performance. Moderator analysis was performed to explore the contributions of underlying factors, such as the characteristics of isometric protocols on its PAPE effects. By doing so, it will ultimately provide critical knowledge informing the design of appropriate isometric protocols in future research and training practice.

## 2 Methods

### 2.1 Literature Search

This systematic review was conducted in accordance with the guidelines outlined in the Cochrane Handbook for Systematic Reviews of Interventions (version 6.5) and the 2020 PRISMA statement (Page et al. 2021). The review protocol was registered on PROSPERO (CRD42024608192) after the literature searches and study screening had been completed, but before data extraction commenced. The eligibility criteria, outcomes of interest, and analysis plan were prespecified prior to data extraction and formal quantitative synthesis. No changes were made to these key protocol components after registration. Before the formal search, one author conducted an informal PubMed search on “isometric training and PAPE/acute performance enhancement.” Studies published up to October 30, 2024 were reviewed, and potentially eligible records were imported into EndNote (version 20; Clarivate Analytics, Philadelphia, PA, USA) for organization and further screening. Subsequently, two independent researchers performed a systematic search of PubMed, Web of Science, EBSCO, and Scopus. English‐language studies published up to October 30, 2024 were considered, with no restriction on the country of origin.

The search strategy incorporated Boolean logic and the following key terms: “isometric”, “maximal voluntary contraction”, “MVC”, “MVIC”, “IMTP”, “conditioning activity”, “acute enhancement”, “acute improvement”, “PAP”, “PAPE”, “post‐activation potentiation”, “post‐activation performance enhancement”, “potentiation”, and “warm‐up”. Full search strings are provided in Supporting Information S1. Manual searches also included (a) screening the reference lists of all included studies and relevant reviews on PAPE and isometric training; (b) examining studies citing the included or similar studies via PubMed; and (c) setting up search alerts to identify newly published studies after the last search through December 10, 2024. No additional eligible studies were identified, and the final inclusion date remained October 30, 2024.

### 2.2 Eligibility Criteria

Three authors developed the eligibility criteria following the PICOS protocol. One author conducted the identification and screening of studies based on titles and abstracts. Two authors independently reviewed the full texts using the inclusion and exclusion criteria (for a precise description, **s**ee Table 1). Any disagreements were initially resolved through discussion between the two independent reviewers, with a third author consulted for dispute resolution.

**TABLE 1:** Study inclusion–exclusion criteria.

Components	Criteria	Inclusion	Exclusion
Population	1	Healthy participants of any sex, age, and training background were eligible. No minimum training‐status threshold was applied.	Special populations, such as patients or participants with injuries or diseases.
Intervention	2	Acute interventions employing isometric contraction, where the muscle‐tendon unit exerts force against a constant load or resistance without changing length, were included. This encompassed both HIMA and PIMA, with no restrictions on intensity, load, or equipment.	Pseudo‐isometrics (e.g., eccentric quasi‐isometrics or functional isometric muscle action (Oranchuk, Storey, Nelson, and Cronin 2019b; Berning et al. 2010)), or other movements that did not strictly maintain a constant muscle length during the intervention.
	3	Isometric contraction was incorporated in complex/contrast training, where the subsequent exercise was considered a performance test.	The performance of the exercise following the isometric contraction was not assessed; studies in which any exercise rather than resting was performed between the performance assessment and isometrics.
Comparator	4	Studies with a control condition (CON), a dynamic isotonic contraction comparator (DYN), or no separate comparator condition were eligible, provided that pre–post acute performance changes could be assessed.	Studies using only nonrelevant comparator conditions without an isometric conditioning activity of interest.
Outcome	5	Studies reporting performance outcomes related to muscular strength (e.g., MVC, peak force, force output, and 1RM), power (peak/mean power), or vertical jump height performance (e.g., countermovement vertical jump height, and squat jump height).	Outcomes not reflecting acute post‐conditioning strength‐, power‐, or jump height performance. Multi‐repetition performance tasks in which endurance capacity could substantially influence performance (e.g., 3RM, 5RM, and repeated jumps) were excluded.
	6	Studies were eligible if they included both a baseline and a post‐conditioning performance assessment, allowing the acute effect of the isometric conditioning activity to be evaluated, with a rest interval of ≤ 60 min.	Studies that did not include a baseline assessment or that performed the post‐test after a rest interval of > 60 min were excluded.
Study design	7	Experimental studies, including randomized or nonrandomized within‐subject/crossover designs, repeated‐measures designs, parallel‐group controlled trials, and other comparative acute designs.	Nonexperimental studies (e.g., reviews, meta‐analyses, conference abstracts, editorials, commentaries, and case reports) or nonacute studies.
Other criteria	8	Peer‐reviewed and published studies in English were included	Studies published in other languages were excluded.
	9	Studies with available full‐text and data for extraction were included.	Studies with unavailable full‐text or original data, or those with no response from authors, were excluded.

Abbreviations: HIMA, holding isometric muscle action; PIMA, pushing isometric muscle action; RM, repetition maximum; MVC, maximal voluntary contraction; RCTs, randomized controlled trials.

### 2.3 Data Extraction

All included studies were randomly assigned to three independent authors for data extraction. The following data were systematically extracted into a pre‐designed Excel spreadsheet (Blazevich and Babault 2019): basic information (i.e., authors and year of publication) (Stone et al. 2008); intervention protocol (i.e., intensity, contraction duration, volume, intervention modality, repetition configuration, and joint angle) (DeRenne and Journal 2010); participant characteristics (i.e., number of participants, age, sex, and strength training experience) (Ebben WPJJoss, 2002); and outcome (i.e., sample sizes, means, and standard deviations for pre‐ and post‐intervention tests of relevant outcome measures). Additionally, mean changes and standard deviations of changes for control and dynamic isotonic contraction groups were extracted, along with the specific time points of post‐tests for all groups. Effect sizes of < 0.2, 0.2–0.49, 0.5–0.79, and ≥ 0.8 were interpreted as trivial, small, moderate, and large, respectively (Cohen 2013).The data extraction files were cross‐checked by the three authors, and any discrepancies were resolved through discussion and consultation with the corresponding author.

If the original data were not reported or were incomplete, the corresponding authors of the included studies were contacted directly. If no response was received (up to January 5, 2025) or if the authors declined to provide the original data, the calculations were performed according to the methods described in Supporting Information S2. When values were presented as figures, data were digitized using a graph digitizer software (WebPlotDigitizer, version 4.8, https://apps.automeris.io/wpd4/).

### 2.4 Coding of the Study

The included studies were categorized according to a predetermined scheme derived from prior reviews relevant to the current topic (Oranchuk et al. 2019a, 2024; Lum and Barbosa 2019) (see Table 2 for details). Contraction duration was calculated as the total number of seconds per intervention session (sustained contraction duration per repetition × repetitions × sets); volume was defined as the intensity‐weighted contraction stimulus within one intervention session (Carvalho et al. 2022). No well‐established definition of training volume exists (Oranchuk et al. 2019a, 2024); session volume was thus calculated using the following equation:

volume=relativeintensity×contractionduration

**TABLE 2:** Coding of studies.

Variable	Sub‐variable	Category
Comparison format		Within the isometric group: pre versus post; Between groups: ISO versus CON and ISO versus DYN.
Participants' characteristics	Sex	Male, female, and mixed
	Strength training experience	< 2 years and ≥ 2 years (for vertical jump and power output outcomes) Strength untrained and strength trained (for muscular strength outcomes)
Intervention variables	Intensity	Maximal and submaximal
	Contraction duration	Short: ≤ 8 s Moderate: 8 < duration < 12 s Long: 12 ≤ duration ≤ 15 s Prolonged: > 15 s
	Volume	Low: ≤ 6 unit
		Moderate: 6 < duration ≤ 10 unit
		High: 10 < duration ≤ 15 unit
		Very high: > 30 unit
	Intervention modality	HIMA and PIMA
	Rep configuration	Single rep and multiple reps
	Joint angle	Knee angle ≤ 90° 90° < knee angle ≤ 140° Unclear
Outcome measurement	Outcome variable	Muscular strength (MVC, PF, and PT) Power output (PPO, MPO) Vertical jump height (CMJ, SJ, and DJ)
	Rest intervals	Short interval: < 4 min Moderate interval: 4 ≤ interval ≤ 8 min Long interval: < 8 interval ≤ 12 min Prolonged interval: > 12 min

Abbreviations: CMJ, counter‐movement jump; CON, control group; DJ, drop jump; DYN, dynamic isotonic contraction group; Sec, second; HIMA, holding isometric muscle actions; ISO, isometric contraction group; MVC, maximal voluntary contraction; Min, minute; Volume unit = relative intensity × contraction duration; one unit represents 1 s of maximal isometric contraction or the equivalent intensity‐weighted contraction stimulus; MPO, mean power output; PIMA, pushing isometric muscle actions; PF, peak force; PT, peak torque; PPO, peak power output; Rep, repetition; SJ, squat jump.

Maximal intensity was coded as 1.0 and submaximal intensity was expressed as a decimal (e.g., 80% MVC or 80% 1RM = 0.80). Accordingly, one volume unit represented 1 s of maximal isometric contraction or the equivalent intensity‐weighted contraction stimulus under submaximal conditions (Details in Supporting Information S3).

### 2.5 Assessment of Risk of Bias and Study Quality

A modified version of the Cochrane Collaboration tool was utilized to assess the risk of bias of the included studies (Higgins 2011), and the quality of the included studies was evaluated using the modified PEDro scale, as adapted by (Brughelli et al. 2008), as a complementary assessment of methodological completeness and reporting quality. We selected this combined approach, but not a single standard tool such as ROB2 or ROBINS‐I, since the included studies were methodologically heterogeneous (e.g., randomized and nonrandomized).

For the modified version of the Cochrane Collaboration tool, two aspects (allocation concealment and blinding of implementers) were excluded due to the challenges of implementation in PAPE studies (Xu et al. 2024). Four PAPE‐specific domains were included to better capture sources of bias that are particularly relevant to acute conditioning activity studies (i.e., cumulative effects bias, measurement bias, sample size bias, and test familiarization) (Xu et al. 2024). Specifically, cumulative effect bias was defined as the potential for repeated post‐CA performance tests to influence subsequent outcomes through carryover effects. This domain was operationalized according to the number and spacing of post‐CA assessments, adapted from a recent PAPE‐specific meta‐analysis (Xu et al. 2024), which was justified with reference to the phosphocreatine recovery time (McMahon and Jenkins 2002; Jones et al. 2007). Measurement bias was evaluated based on the reporting of reliability statistics. Sample size bias was assessed according to whether an a priori sample size estimation was performed and correctly described. Test familiarization was evaluated based on whether familiarization sessions were conducted and, if so, how many. Detailed descriptions regarding the criteria of assessment can be found in Supporting Information S4.

In terms of the modified PEDro scale (Brughelli et al. 2008), the scale assesses whether (Blazevich and Babault 2019) eligibility criteria were specified (Stone et al. 2008); participants were randomly allocated (DeRenne and Journal 2010); interventions were clearly described (Ebben WPJJoss, 2002); baseline similarity between groups was established (Mihalik et al. 2008); a control condition was included (Rassier et al. 2000); outcome measures were clearly defined (Sale 2002); the assessments had practical relevance (Sale DJBjosm 2004); the intervention duration had practical relevance (Xu et al. 2024); between‐group statistical analyses were appropriate; and (Seitz and Haff 2016) measures of variability were reported. The instrument includes 10 items, yielding a total score of 0–20, with each item scored as 0 = clearly no, 1 = maybe, and 2 = clearly yes (detailed information can be accessed in Supporting Information S5). Initial discussions on the modified Cochrane Collaboration tool and modified PEDro scale were conducted by three authors. Evaluations were then carried out independently, adhering to pre‐established uniform criteria, with discrepancies resolved through discussion.

### 2.6 Statistical Analysis

#### 2.6.1 Three‐Level Meta‐Analysis

In the context of PAPE studies, it is common to encounter multiple performance assessments conducted at different time points within the same study (Krzysztofik, Wilk, et al. 2023; Koźlenia and Domaradzki 2024), which introduce dependencies among effect sizes that can lead to biased estimates and inflated statistical significance if not properly accounted for (Jukic et al. 2023; Assink and Wibbelink 2016).Therefore, a three‐level meta‐analysis (multilevel model) was performed using *R* (Ver. 2024.12.0 + 467; Vienna, Austria) and the *metafor* package (Assink and Wibbelink 2016), and utilizing the open‐source *R* code developed by Jukic et al. 2023. This approach accommodates the nested structure of the data by modeling effect sizes (Level 1) within studies (Level 2), which are further nested within the overall population of studies (Level 3). By doing so, the model effectively decomposes the observed variance into three components: sampling variance (Level 1), within‐study variance (Level 2), and between‐study variance (Level 3) (Cheung 2014, 2019). The model parameters were estimated using the restricted maximum likelihood (REML) method and subsequently cross‐validated by the maximum likelihood (ML) method to ensure result stability. Individual coefficients in all models, along with their corresponding confidence intervals (CIs), were tested using a t‐distribution (Jukic et al. 2023). Dependency among effect sizes was therefore addressed through the multilevel modeling framework described above, and cluster‐robust variance estimation was not additionally applied.

#### 2.6.2 Moderator Analysis and Meta‐Regression

To examine variables potentially influencing the PAPE response to ISO, moderators were selected a priori based on conceptual relevance to the outcomes and data availability, with only those supported by at least eight effect sizes included (HJTJCJCMLTPMWV 2024). Levels with insufficient or unclear data (e.g., mixed‐sex groups and unclear joint angles) were excluded. Full descriptions of included moderators are provided in Supplementary Material 9. Models with and without intercepts were used to evaluate moderator levels relative to the reference category. Moderator effects were reported with 95% confidence intervals (CIs) and, where applicable, 95% prediction intervals (PIs).

In order to explore the relationship between different continuous variables (contraction duration, volume, and rest interval) and isometric‐induced PAPE, the linear and nonlinear meta‐regressions were conducted within the ISO group (pre vs. post comparison). All models were evaluated using both linear and various nonlinear meta‐regression models, including simple linear, cubic polynomial, restricted cubic spline, natural cubic spline, and thin plate spline models (Harrell 2001). Model selection was informed not only by goodness‐of‐fit indices but also theoretical interpretability (Nuzzo et al. 2024). Specifically, based on prior literature and the physiological nature of PAPE, a nonlinear, potentially inverted‐U‐shaped dose–response relationship was expected a priori (Xu et al. 2024; Tillin and Bishop 2009). Therefore, spline‐based nonlinear models were prioritized theoretically. We then extracted the peak points (the maximum effect size within the fitted profile) (Xu et al. 2024). Among all nonlinear models assessed in this study, the natural cubic spline provided the best goodness‐of‐fit. All regression analyses were conducted using the *metafor* package and subsequently visualized with the *ggplot2* package in *R*.

#### 2.6.3 Sensitivity Analysis and Publication Bias

In order to ensure the robustness of the estimates, *leverage, outlier,* and *influential* diagnostics were performed for all meta‐analysis models by calculating *hat values, Cook's distance,* and *studentized residuals,* using the open‐source *R* code reported by (Jukic et al. 2023). Cases were flagged as potential outliers if their hat values and Cook's distance exceeded three times their respective means and if their studentized residuals had absolute values greater than 3. Subsequently, the three‐level meta‐analysis was repeated after excluding these outliers. Additionally, a range of correlations between the outcomes were imputed (*ρ* = 0.4–0.8) to examine the stability of the estimates.

The risk of publication bias was assessed when the number of studies for each outcome exceeded the recommended threshold of 10 (Page et al. 2019; Sterne et al. 2011). Publication bias was examined separately for within‐study (Level 2) and between‐study (Level 3, averaging the effect sizes within individual studies) assessments, using funnel plots and Egger's test. A *p*‐value < 0.05 indicated the presence of publication bias (Peters et al. 2008; Egger et al. 1997).

#### 2.6.4 Certainty of Evidence Assessment

The **GRADE** framework was used to assess the overall certainty of the evidence, with certainty levels ranging from very low to very high (Schünemann et al. 2019; Guyatt et al. 2011). The GRADE approach evaluates five key domains to determine evidence certainty: risk of bias, inconsistency of results, indirectness of evidence, imprecision, and publication bias. Initial assessments were conducted independently by two authors, with a subsequent review by a third author to ensure consensus and resolve any discrepancies.

## 3 Results

### 3.1 Study Selection and Study Characteristics

**FIGURE 1:**
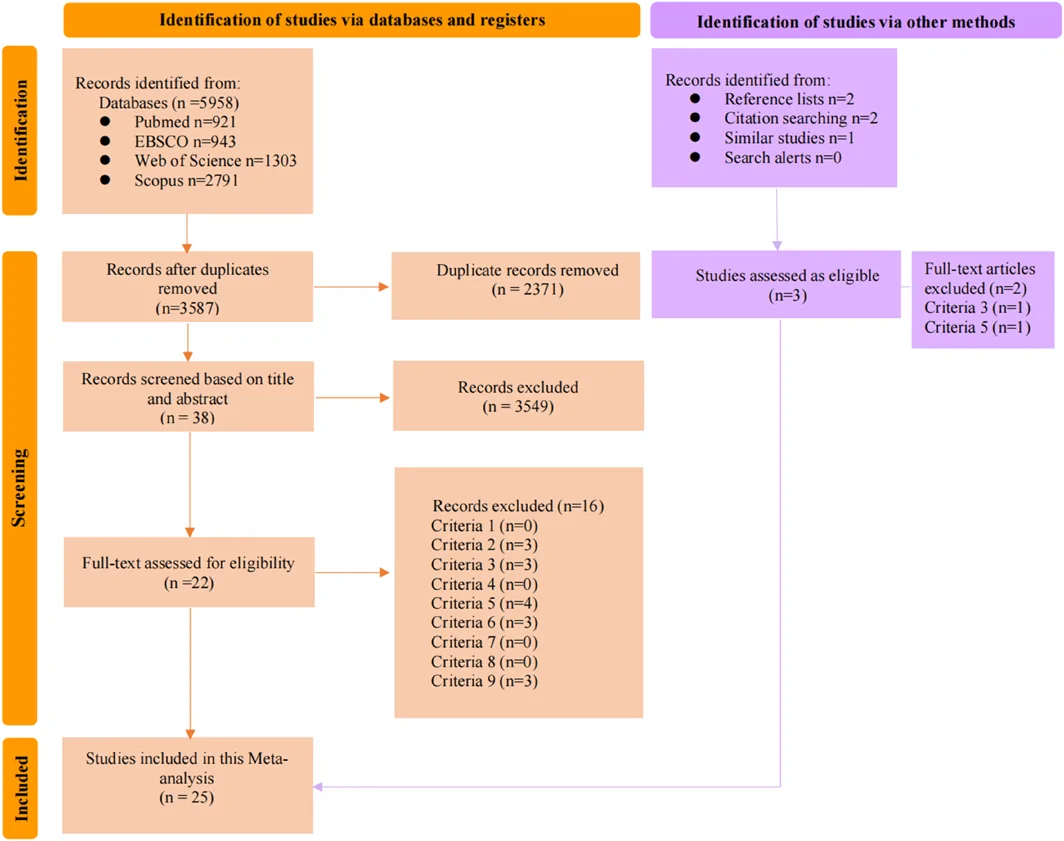
Flow diagram of study selection.

A total of 5958 studies were retrieved through database screening: PubMed (*n* = 921), EBSCO (*n* = 943), Web of Science (*n* = 1303), and Scopus (*n* = 2791). After removing 2371 duplicates using EndNote software, 3587 studies were excluded based on their titles and abstracts. A total of 38 studies were deemed eligible for further evaluation. Following full‐text screening, 25 studies were included in the final meta‐analysis, with 22 identified through database searches and 3 through other methods (Koźlenia and Domaradzki 2023a, 2023b; Rixon et al. 2007; Ino et al. 2024; Downey et al. 2022; Tsoukos et al. 2016; D PIPER et al. 2020; French et al. 2003; Herring et al. 2021; Spieszny et al. 2022; Koźlenia and JJFiP 2023; Vargas‐Molina et al. 2021; Batista et al. 2011; Koźlenia and Domaradzki 2024; Skurvydas et al. 2019; Esformes et al. 2011; Garcia et al. 2020; Hernández‐Trujillo et al. 2024; Jarosz et al. 2024; Koźlenia 2024; Krzysztofik, Spieszny, et al. 2023; Lima et al. 2014; Montano et al. 2021; Robbins and Docherty 2005; Tsolakis et al. 2011) (Figure 1). The 25 studies enrolled 782 participants (601 men, 181 women; mean age 23.5 ± 5.1 y, range 17–52). Nineteen samples were strength‐trained, four untrained, and two mixed. Most studies targeted the lower limb (24/25) (e.g., squat/leg‐press/knee extension/plantar flexion/hip‐hinge); three targeted the upper limb (bench press). Intensities ranged 20%–100% MVC (18 maximal; 8 submaximal). Contraction duration spanned 5–60 s; volume 5–60 units; multiple repetitions were more common (19 vs. Eight single). PIMA was used in 18 studies and HIMA in 6. Reported knee angle range was 60°–140°. Vertical jump was the dominant outcome (20 studies: CMJ *n* = 17; SJ *n* = 3; DJ *n* = 2), with strength (Sale 2002) and power (Ebben WPJJoss, 2002) being less frequent. Post‐intervention intervals ranged 0–60 min, whereas most observations clustering in the short–moderate windows (Tables 3 and 4).

**TABLE 3:** Summary of participants' characteristics.

Study	Participants	Strength training experience	Sample	Age
Batista et al. (2011)	8 track and field athletes 7 hypertrophy‐oriented trainers And 8 health‐oriented trainers	≥ 2 years ≥ 2 years < 2 years	23M	23.3 ± 3.7
Downey et al. (2022)	Trained men	< 2 years	14M 10 F	23.3 ± 4.4
Esformes et al. (2011)	Rugby players	≥ 2 years	10M	20.4 ± 0.8
French et al. (2003)	Track and field athletes	≥ 2 years	14M	23 ± 5.7
Garcia et al. (2020)	Trained men	≥ 2 years	13M	24 ± 3.14
Hernández‐Trujillo et al. (2024)	Men and women	< 2 years	30M 30 F	46.4 ± 5.5
Herring et al. (2021)	Trained men and women	≥ 2 years	15M 15 F	M22.3 ± 2.5 F22 ± 1.7
Ino et al. (2024)	Jump athletes	< 2 years	14M	21.1 ± 1.4
Jarosz. (2024)	Soccer players	≥ 2 years	15M	26.9 ± 4.2
Koźlenia and Domaradzki. a (2023)	Trained women	≥ 2 years	41F	21.08 ± 1.42
Koźlenia and Domaradzki. b (2023)	Trained men	≥ 2 years	41M	19.6 ± 1.5
Koźlenia and Domaradzki. c (2024)	Trained men	≥ 2 years	45M	21.6 ± 2.4
Koźlenia and Domaradzki. d (2023)	Trained men and women	≥ 2 years	30M 15 F	M21.2 ± 1.7 F21.2 ± 2
Koźlenia. e (2024)	Trained men and women	≥ 2 years	63M 42 F	20–27
Krzysztofik et al. (2023)	Handball players	≥ 2 years	12M	20.8 ± 2.2
Lima et al. (2014)	Inactive men	< 2 years	23M	23.5 ± 4
Montano et al. (2021)	Trained men	≥ 2 years	15M	24.7 ± 3.5
Piper et al. (2020)	Trained men and women	< 2 years	10M 3 F	20 ± 2
Rixon et al. (2007)	1 track and field athlete 1 bodybuilder 14 men and 14 women	< 2 years	15M 15 F	23.3 ± 2.7
Robbins and Docherty. (2005)	Trained men	< 2 years	16M	University‐aged
Skurvydas et al. (2019)	Physically active	< 2 years	120M	University‐aged
Spieszny et al. (2022)	Handball and soccer players	≥ 2 years	31M	19 ± 2
Tsolakis et al. (2011)	Fencers	< 2 years	13M 10 F	M21.8 ± 3.7 F22.7 ± 4.8
Tsoukos et al. (2016)	Track and field athletes	≥ 2 years	14M	27.1 ± 7.0
Vargas‐Molina et al. (2021)	Trained men	≥ 2 years	18M	25.8 ± 2.7

Abbreviations: M, male; F, female.

**TABLE 4:** Summary of study characteristics.

Study	ISO group		Comparison group		Outcomes	Rest interval
	Intervention	Sets/Duration/Intensity	Intervention	Sets/Reps/Intensity		
Batista et al. (2011)	ISO leg press	1/5s/100%MVC 3/5s/100%MVC	CON: rest	NA	CMJ	4 min
Downey et al. (2022)	ISO squat ISO bench press	1/7s/85%1RM	CON: rest	NA	CMJ	0–8 min
Esformes et al. (2011)	ISO bench press	1/7s/100%MVC	DYN: bench press	1/3/3RM	PP, PF	12 min
French et al. (2003)	ISO knee extension	3/3s/100%MVC 3/5s/100%MVC	CON: rest	NA	DJ, MF, PT	0 min
Garcia et al. (2020)	ISO squat ISO knee extension	3/5s/100%MVC 3/5s/100%MVC	No comparison	NA	SJ, MP, PP	3, 7 min
Hernández‐Trujillo et al. (2024)	ISO knee extension ISO hip abduction ISO hip extension ISO knee flexion ISO knee extension	3/5s/20‐30%MVC 3/5s/45‐55%MVC 3/5s/100%MVC	CON: rest	NA	CMJ, MVC	3 min
Herring et al. (2021)	ISO mid‐thigh pull	3/3–5s/100%MVC	CON: rest	NA	CMJ	4–7 min
Ino et al. (2024)	ISO plantar flexion	1/6s/100%MVC	CON: rest	NA	DJ	10 s
Jarosz. (2024)	ISO squat	1/5s/100%MVC	No comparison	NA	CMJ	1 min
Koźlenia and Domaradzki. a (2023)	ISO squat	3/4s/70%1RM 3/5s/100%MVC	CON: treadmill run	4 min × 6 km/h	CMJ	3, 5, 7, 9 min
Koźlenia and Domaradzki. b (2023)	ISO squat	3/5s/100%MVC	CON: treadmill run	6 min × 6 km/h	CMJ	3, 5, 7, 9 min
Koźlenia and Domaradzki. c (2023)	ISO squat	3/4s/70%1RM	CON: treadmill run	4 min × 6 km/h	CMJ	1, 3, 5, 7.9 min
Koźlenia and Domaradzki. d (2023)	ISO squat	3/4s/70%1RM	No comparison	NA	CMJ	1, 3, 5, 7.9 min
Koźlenia. e (2024)	ISO squat	3/4s/70%1RM	CON: light treadmill activity	NA	CMJ, SJ	3, 5, 7, 9 min
Krzysztofik et al. (2023)	ISO squat	3/3s/100%MVC	CON: rest	NA	CMJ	4, 8, 12 min
Lima et al. (2014)	ISO knee extension	1/5s/100%MVC	No comparison	NA	IPT	3 min
Montano et al. (2021)	25% lower limb height ISO deadlift 75% lower limb height ISO deadlift	3/5s/100%MVC	No comparison	NA	PF	4 min
Piper et al. (2020)	ISO squat	3/3s/100%MVC	DYN: squat CON: walk	3/5/87%1RM 4 min	CMJ	20 s, 4, 8, 12, 16, 20 min
Rixon et al. (2007)	ISO squat	3/3s/100%MVC	DYN: half squat	1/3/3RM	CMJ, PP	3 min 0 min
Robbins and Docherty (2005)	ISO squat	1/7s/100%MVC	CON: rest	NA	CMJ	4 min
Skurvydas et al. (2019)	ISO knee extension	5/1s/100%MVC 1/5s/100%MVC 15/1s/100%MVC 1/10s/100%MVC 6/5s/100%MVC 1/30s/100%MVC 1/60s/50%MVC 1/60s/100%MVC 12/5s/100%MVC	No comparison	NA	MVC	0, 5, 10, 30, 60 min
Spieszny et al. (2022)	ISO squat	3/3s/100%MVC	CON: treadmill run	6.5 min × 5 km/h	CMJ, SJ	4, 8 min
Tsolakis et al. (2011)	ISO bench press, ISO leg press	3/3s/100%MVC	No comparison	NA	PP	0, 4, 8, 12 min
Tsoukos et al. (2016)	ISO squat (knee angle 140°) ISO squat (knee angle 90°)	3/3s/100%MVC	CON: rest	NA	CMJ	15 s, 3, 6, 9, 12 min
Vargas‐Molina et al. (2021)	ISO squat	1/4s/75%1RM	DYN: squat	1/3/75%1RM	CMJ	4 min

Abbreviations: CMJ, countermovement jump; ISO, isometric contraction; CON, control; DYN, dynamic isotonic contraction; MVC, maximal voluntary contraction; NC, no comparison; NA, not applicable; RM, repetition maximum; PP, peak power; PF, peak force; DJ, drop jump; MF, mean force; PT, peak torque; SJ, squat jump; MP, mean power; IPT, isometric peak torque; min, minute; s, second; km, kilometer; h, hour.

### 3.2 Risk of Bias and Study Quality

**FIGURE 2:**
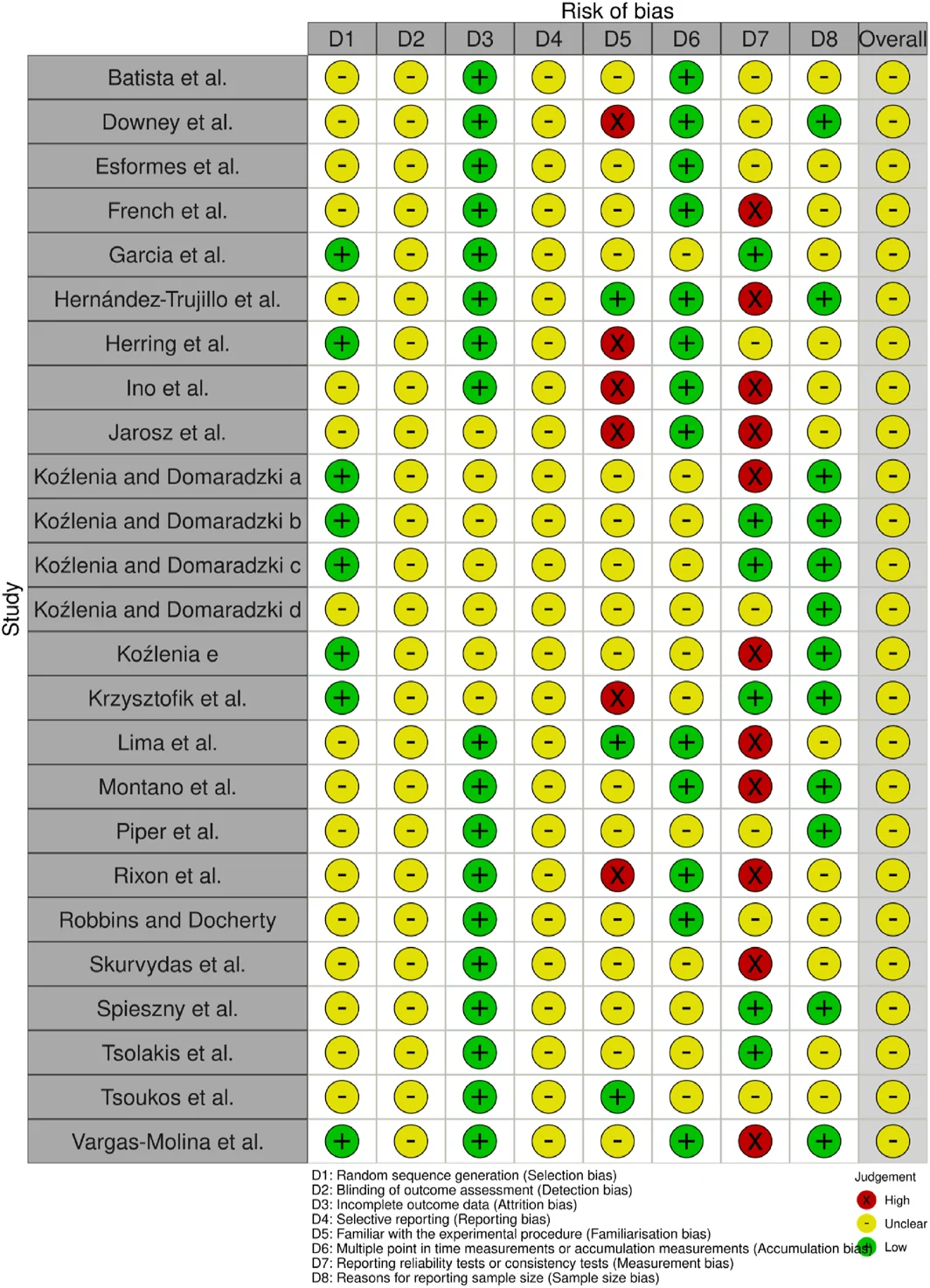
Risk of bias summary of included studies.

Randomization was reported in all studies but detailed in only 8 studies (low risk); no study blinded testers. Risks were frequently unclear for attrition (7 studies), familiarization (22/25 with ≤ 1 or no session), multi‐time‐point assessments (Boullosa 2021), and measurement reliability (only 6 provided multiple indices). Twelve studies reported a priori sample‐size estimation; 13 were unclear despite ≥ 10 participants (Figure 2) (Detailed descriptions of the risk of bias assessment can be found in supplementary material 9). The modified PEDro scores for the studies included in this review ranged from 14 to 20, with a mean score of 16.8. Overall, risk of bias was rated as high or unclear across several domains, despite generally adequate reporting of interventions and outcome measures (Table 5).

**TABLE 5:** Methodological quality for the included studies using the modified PEDro.

Study	IC	RA	ID	BS	CG	OD	AU	DI	SA	VM	Total
Batista et al. (2011)	2	2	2	2	2	2	2	2	2	2	20
Downey et al. (2022)	2	2	2	1	2	2	2	2	2	2	19
Esformes et al. (2011)	2	2	2	1	2	2	2	2	2	2	19
French et al. (2003)	2	2	2	1	2	2	2	2	2	2	19
Garcia et al. (2020)	2	2	2	2	0	2	2	2	2	2	18
Hernández‐Trujillo et al. (2024)	2	2	2	2	2	2	2	2	2	2	20
Herring et al. (2021)	2	2	2	1	2	2	2	2	2	2	19
Ino et al. (2024)	2	2	2	1	2	2	2	2	2	2	19
Jarosz. (2024)	2	0	2	0	0	2	2	2	2	2	14
Koźlenia and Domaradzki. a (2023)	2	2	2	1	2	2	2	2	2	2	19
Koźlenia and Domaradzki. b (2023)	2	2	2	1	2	2	2	2	2	2	19
Koźlenia and Domaradzki. c (2023)	2	2	2	2	2	2	2	2	2	2	20
Koźlenia and Domaradzki. d (2023)	2	0	2	0	0	2	2	2	2	2	14
Koźlenia. e (2024)	2	2	2	2	2	2	2	2	2	2	20
Krzysztofik et al. (2023)	2	2	2	2	2	2	2	2	2	2	20
Lima et al. (2014)	2	0	2	0	0	2	2	2	2	2	14
Montano et al. (2021)	2	2	2	1	0	2	2	2	2	2	17
Piper et al. (2020)	2	2	2	1	2	2	2	2	2	2	18
Rixon et al. (2007)	2	1	2	0	2	2	2	2	2	2	17
Robbins and Docherty (2005)	2	2	2	1	2	2	2	2	2	2	19
Skurvydas et al. (2019)	2	2	2	1	0	2	2	2	2	2	17
Spieszny et al. (2022)	2	2	2	1	2	2	2	2	2	2	19
Tsolakis et al. (2011)	2	2	2	1	2	2	2	2	2	2	19
Tsoukos et al. (2016)	2	2	2	1	2	2	2	2	2	2	19
Vargas‐Molina et al. (2021)	2	2	2	1	2	2	2	2	2	2	19

Abbreviations: AU, assessments were practically useful; BS, groups were tested for similarity at baseline; CG, a control condition was included; DI, intervention duration was practically useful; IC, inclusion criteria were clearly stated; ID, interventions were clearly defined; OD, outcome variables were clearly defined; RA, participants were randomly allocated to groups; SA, between‐group statistical analyses were appropriate; VM, point measures of variability were provided; 0, clearly no; 1, maybe; 2, clearly yes.

### 3.3 Publication Bias and Certainty Assessment

**FIGURE 3:**
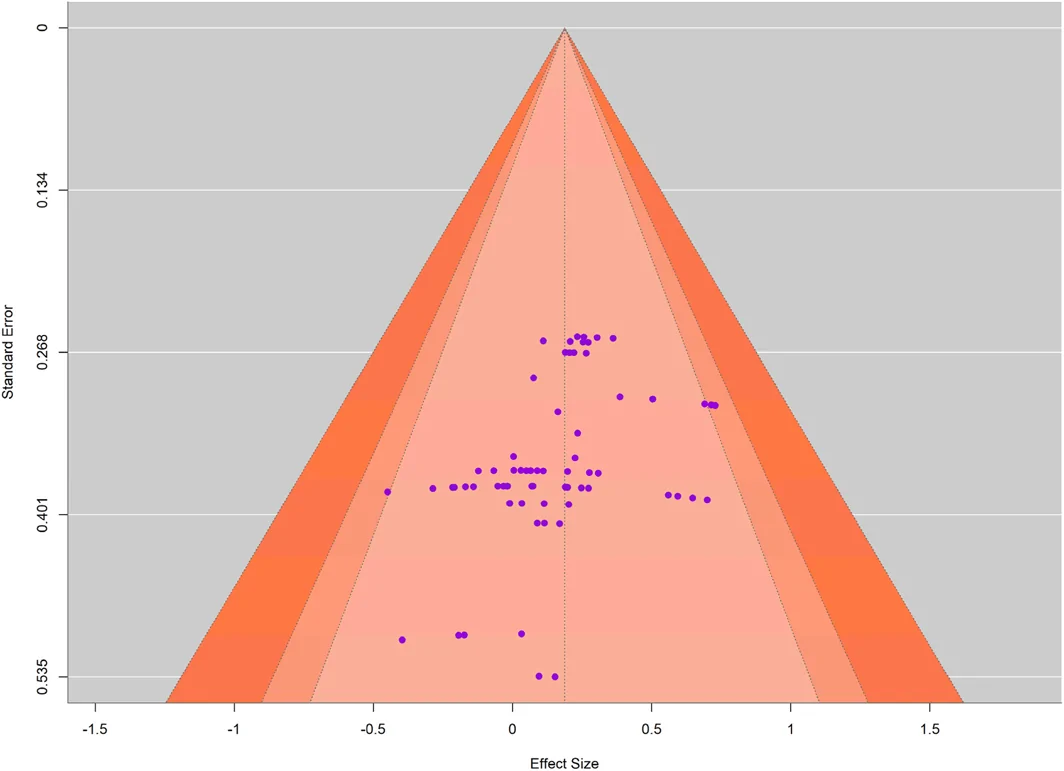
Funnel plot visualization of Level 2 publication bias within the ISO group on vertical jump height.

**FIGURE 4:**
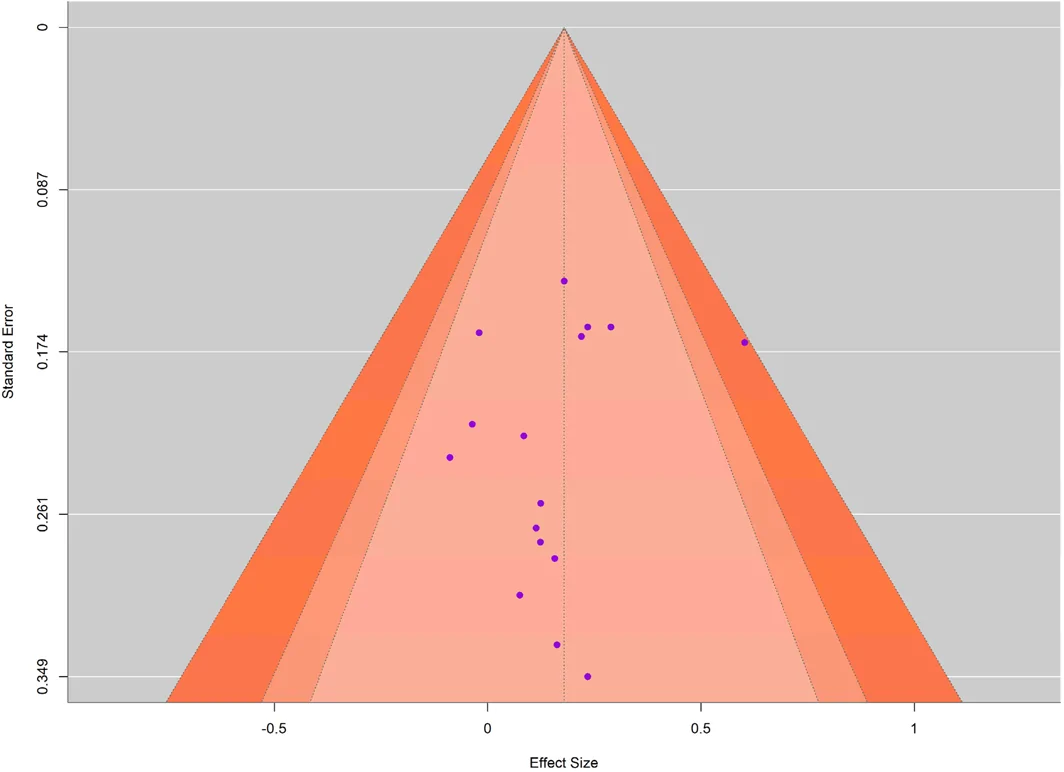
Funnel plot visualization of Level 3 publication bias within the ISO group on vertical jump height.

**FIGURE 5:**
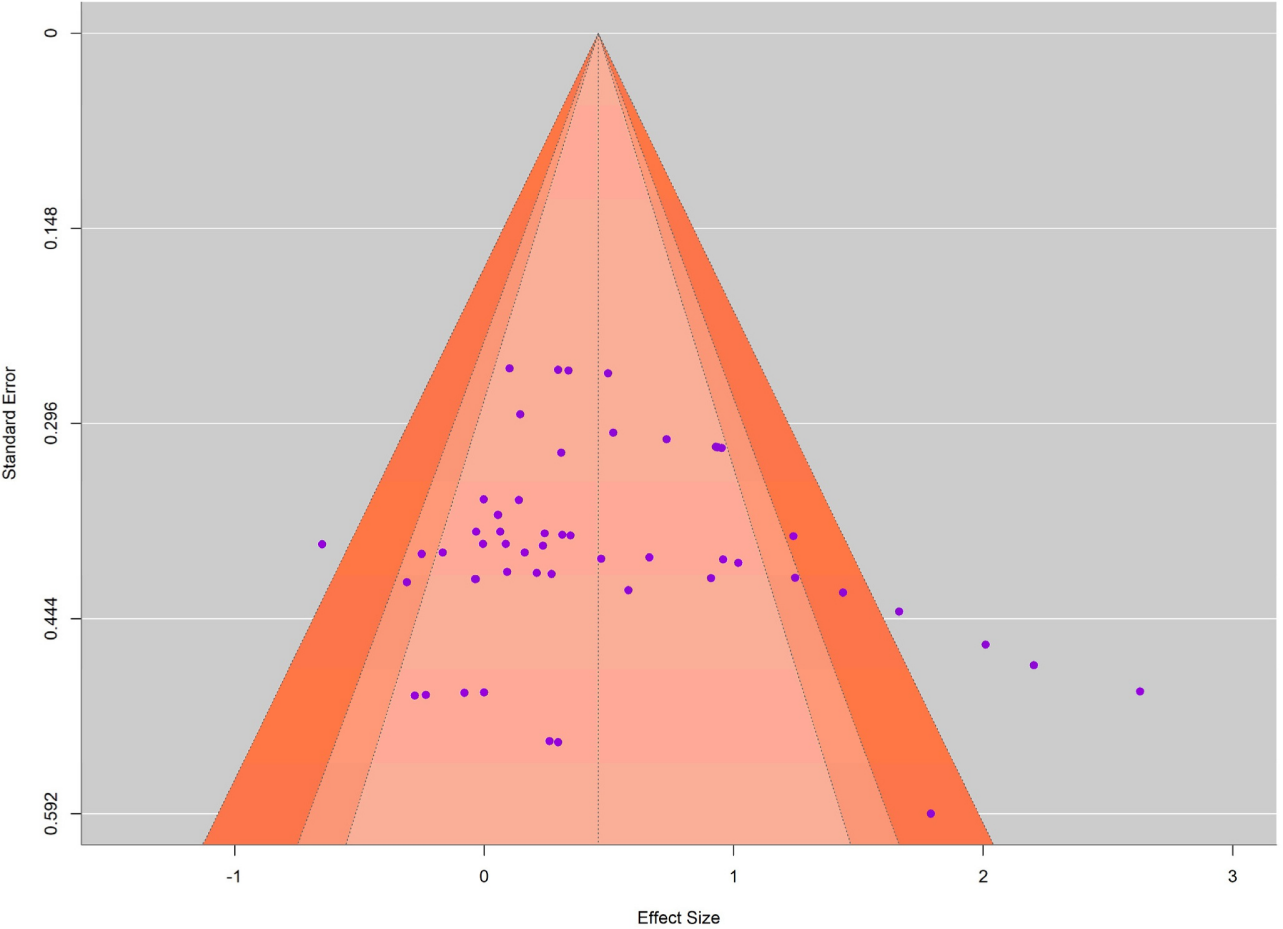
Funnel plot visualization of Level 2 publication bias between the ISO and CON groups on vertical jump height.

**FIGURE 6:**
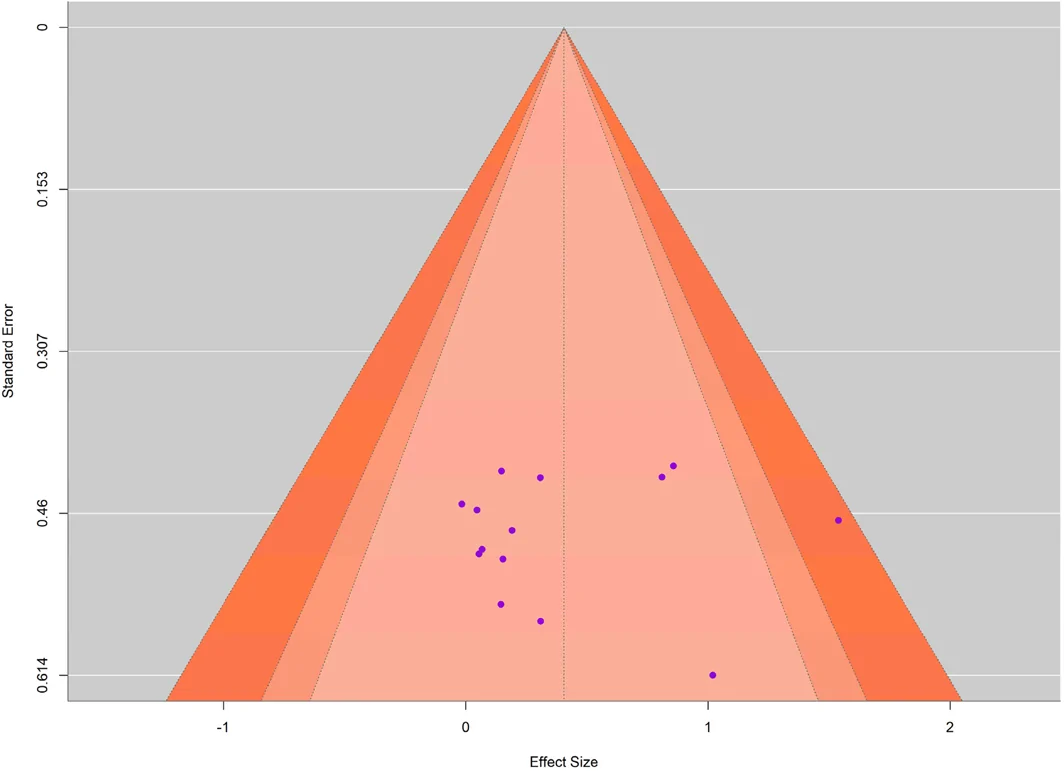
Funnel plot visualization of Level 3 publication bias between the ISO and CON groups on vertical jump height.

For pre versus post comparison within the isometric group on the vertical jump height, Egger's test showed that a significant risk of publication bias was detected at Level 2 (within‐study variance, *p* < 0.05), whereas no significant risk of publication bias was detected at Level 3 (between‐study variance, *p* = 0.365). For isometric versus control group comparison on the vertical jump height, Egger's test showed that no significant risk of publication bias was detected at both Level 2 (within‐study variance, *p* = 0.818) and Level 3 (between‐study variance, *p* = 0.513), and the funnel‐plot indicated a seemingly symmetrical distribution based on visual inspection (Figure 3, 4, 5, 6). Evidence of overall certainty for all comparisons and outcomes was low (See table 6.1‐6.6 in Supporting Information S8).

### 3.4 Results of Meta‐Analysis

#### 3.4.1 Main Effect

**FIGURE 7:**
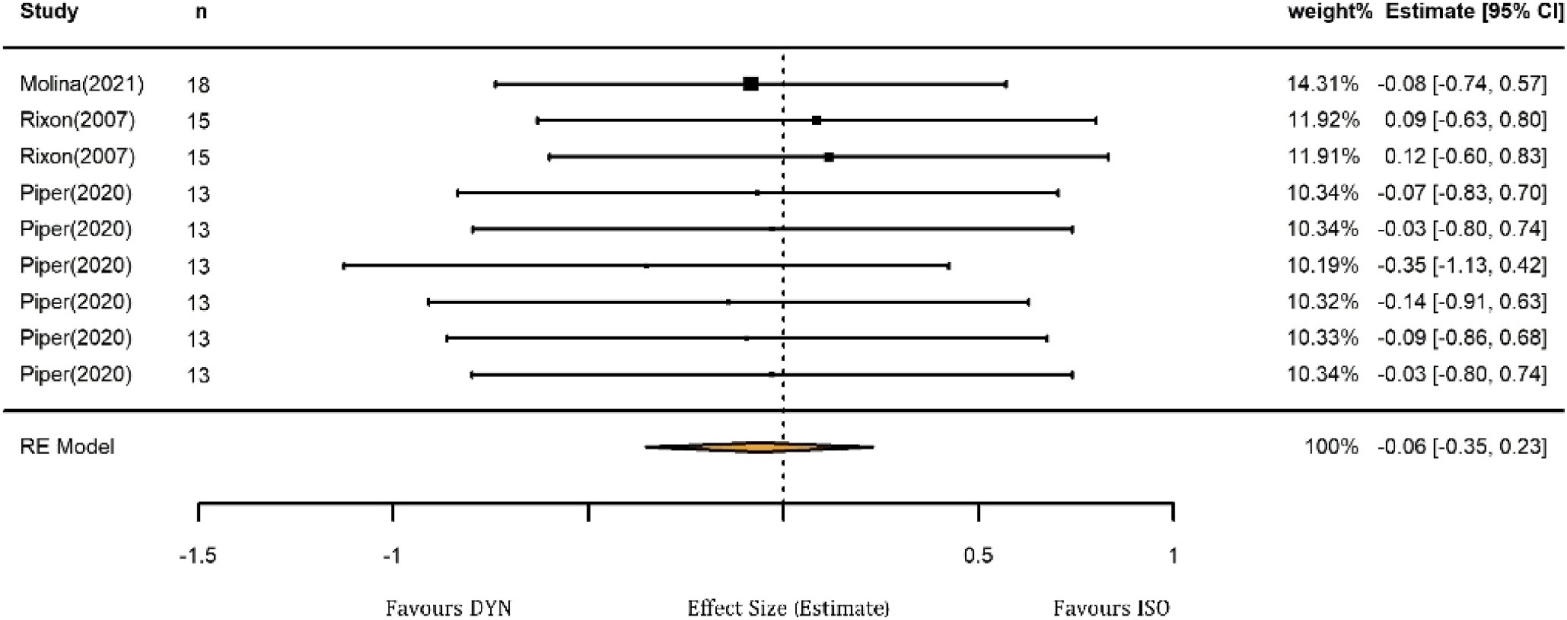
DYN group versus ISO group on vertical jump height. *n*, sample size; CI, confidence interval; DYN, dynamic isotonic contraction group; ISO, isometric contraction group.

Compared to the control group, a significantly greater effect on the vertical jump height in the isometric group was observed with a small effect size (ES = 0.406, SE = 0.127, *t* = 3.204, df = 52, *p* = 0.002, 95% CI [0.152, 0.660], 95% PI [−0.560, 1.372]). Substantial heterogeneity was detected across studies (Q (df = 52) = 125.187, *p* < 0.0001), with a total I^2^ value of 60.65% (I^2^‐level 2 = 18.64%, I^2^‐level 3 = 42.01%), indicating that a significant portion of variance in effect sizes was due to between‐study differences. After excluding four influential effect sizes (Ino et al. 2024; Downey et al. 2022; Tsoukos et al. 2016; Koźlenia 2024) identified through sensitivity analysis, the revised model remained significant, with a slightly smaller effect size (ES = 0.349, SE = 0.110, *t* = 3.175, df = 48, *p* = 0.003, 95% CI [0.128, 0.569], 95% PI [−0.365, 1.062], I^2^‐level 2 = 7.46%, I^2^‐level 3 = 37.58%). The revised model was retained due to the narrower confidence interval providing a more robust estimation. However, no significant difference in vertical jump height between the isometric contraction and the dynamic isotonic contraction was observed (ES = −0.061, SE = 0.126, *t* = −0.483, df = 8, *p* = 0.642, 95% CI [−0.352, 0.230], 95% PI [−0.352, 0.230]) (Figure 7). No heterogeneity was detected across studies (Q (df = 8) = 1.002, *p* = 0.998), with a total I^2^ value of 0%. Sensitivity analysis identified no influential studies.

A significant improvement in vertical jump height was observed within the isometric contraction group with a trivial effect size (ES = 0.157, SE = 0.054, *t* = 2.912, df = 69, *p* = 0.005, 95% CI [0.049, 0.264], 95% PI [−0.114, 0.427]). No significant heterogeneity was detected across studies (Q (df = 69) = 47.215, *p* = 0.979), and the total I^2^ value was 11.65% (I^2^‐level 2 = 0%, I^2^‐level 3 = 11.65%), indicating a low heterogeneity. After excluding four influential effect sizes (Ino et al. 2024; Tsoukos et al. 2016; Herring et al. 2021) identified through sensitivity analysis, the revised model showed a significant effect with a slightly larger effect size (ES = 0.180, SE = 0.051, *t* = 3.518, df = 65, *p* = 0.0008, 95% CI [0.078, 0.282], 95% PI [−0.041, 0.401], I^2^‐level 2 = 0%, I^2^‐level 3 = 7.67%). The revised model was retained due to the narrower confidence interval providing a more robust estimation.

No between group comparison analysis on power output was performed due to the limited number of studies. No significant effect on power output was observed within the isometric contraction group (ES = −0.130, SE = 0.315, *t* = −0.412, df = 26, *p* = 0.684, 95% CI [−0.777, 0.517], 95% PI [−1.549, 1.289]). No significant difference in muscular strength performance between the isometric contraction and the control groups was observed (ES = 0.542, SE = 0.336, *t* = 1.615, df = 9, *p* = 0.141, 95% CI [−0.217, 1.301], 95% PI [−1.010, 2.095]). No significant effect on muscular strength was observed within the isometric contraction group (ES = 0.233, SE = 0.309, *t* = 0.754, df = 57, *p* = 0.454, 95% CI [−0.386, 0.852], 95% PI [−2.082, 2.548]) (Detailed results are in Supporting Information S9).

#### 3.4.2 Moderator Analysis

**FIGURE 8:**
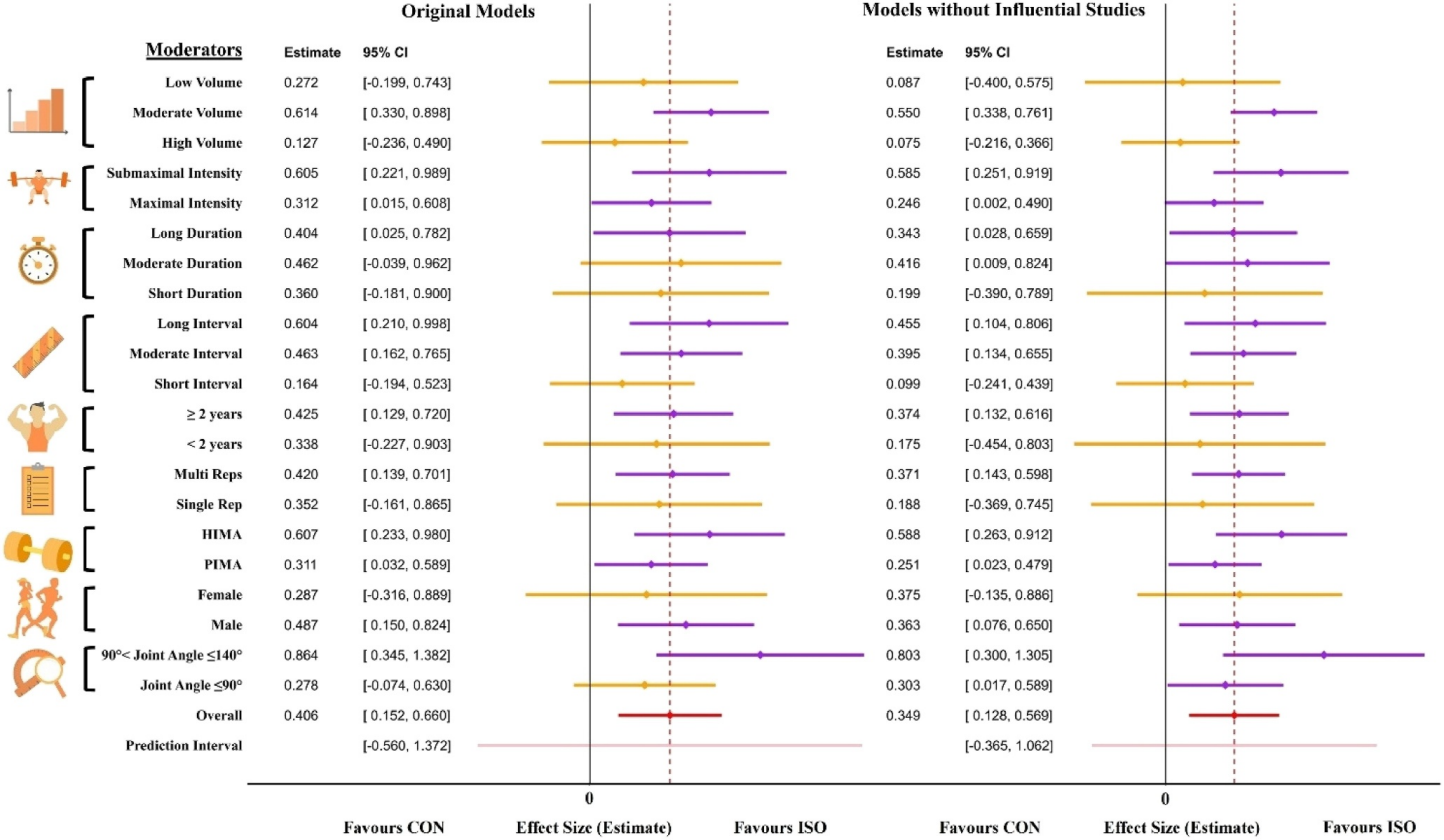
CON group versus ISO group on vertical jump height. CI, confidence interval; Rep, repetition; HIMA, holding isometric muscle actions; PIMA, pushing isometric muscle actions; CON, control group; ISO, isometric contraction group.

**FIGURE 9:**
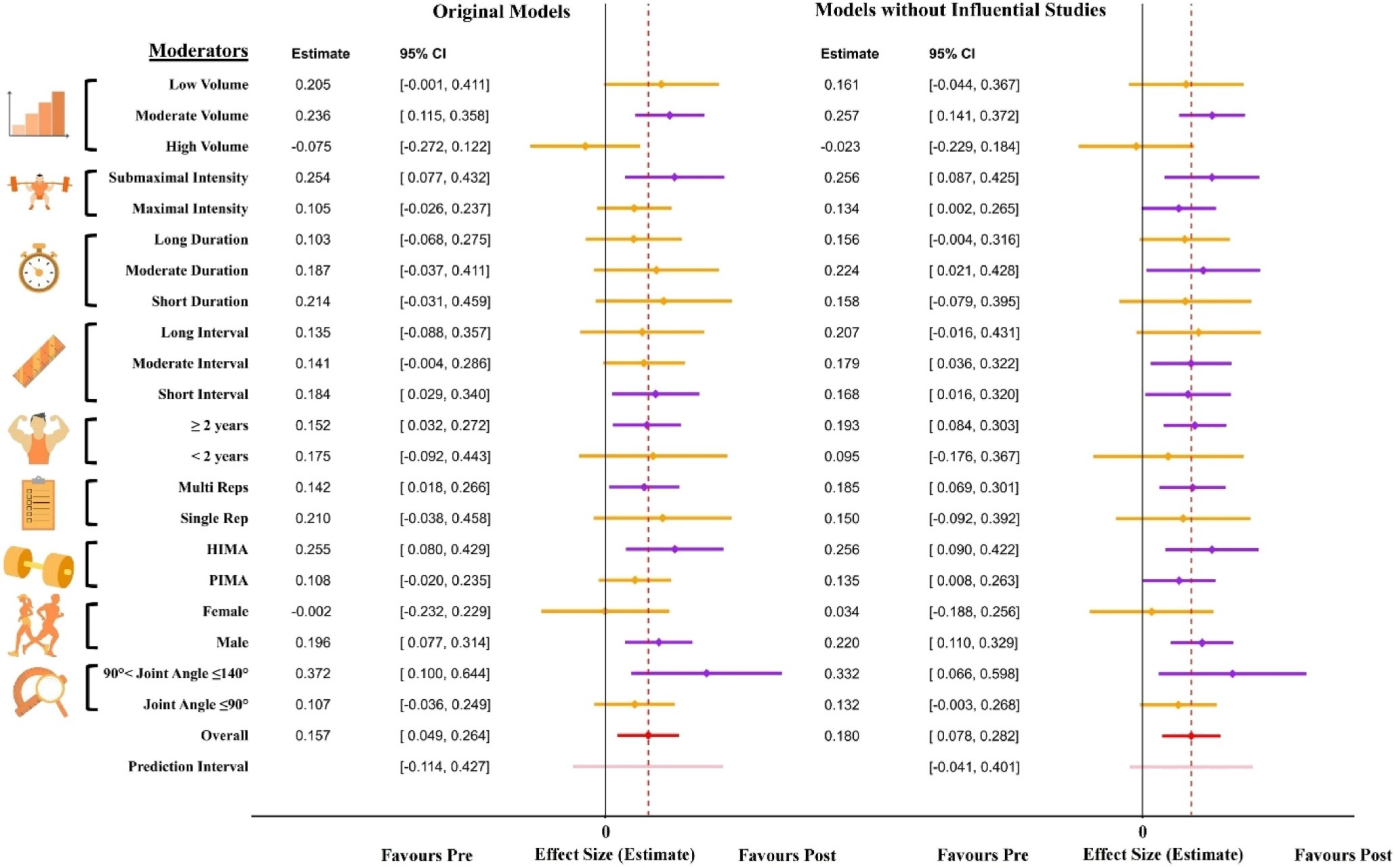
Pre versus post within the ISO group on vertical jump height. CI, confidence interval; Rep, repetition; HIMA, holding isometric muscle actions; PIMA, pushing isometric muscle actions; Pre, pre‐test; Post, post‐test.

For vertical jump height, subgroup‐specific pooled effects estimated from the no‐intercept models suggested significant effects across multiple moderator levels in both the between‐group (ISO vs. CON) and within‐group (pre‐vs. post‐ISO) analyses, including sex, strength training experience, intensity, contraction duration, volume, rest interval, repetition configuration, intervention modality, and joint angle (Figures 8 and 9). Regarding the with‐intercept models, no moderator showed a significant between‐level effect in the within‐group comparison. In the between‐group comparison, the joint angle remained a significant moderator (*F* = 4.325, *p* = 0.043), with greater effects observed for knee angles between 90° and 140° than for angles ≤ 90° (difference = 0.500, 95% CI [0.015, 0.984]). The volume also showed a significant effect (*F* = 4.499, *p* = 0.017), although individual contrasts did not reach statistical significance. All other moderators were not statistically significant in the with‐intercept models (Details in Supporting Information [Link], [Link], [Link] and S9).

#### 3.4.3 Meta‐Regression

**FIGURE 10:**
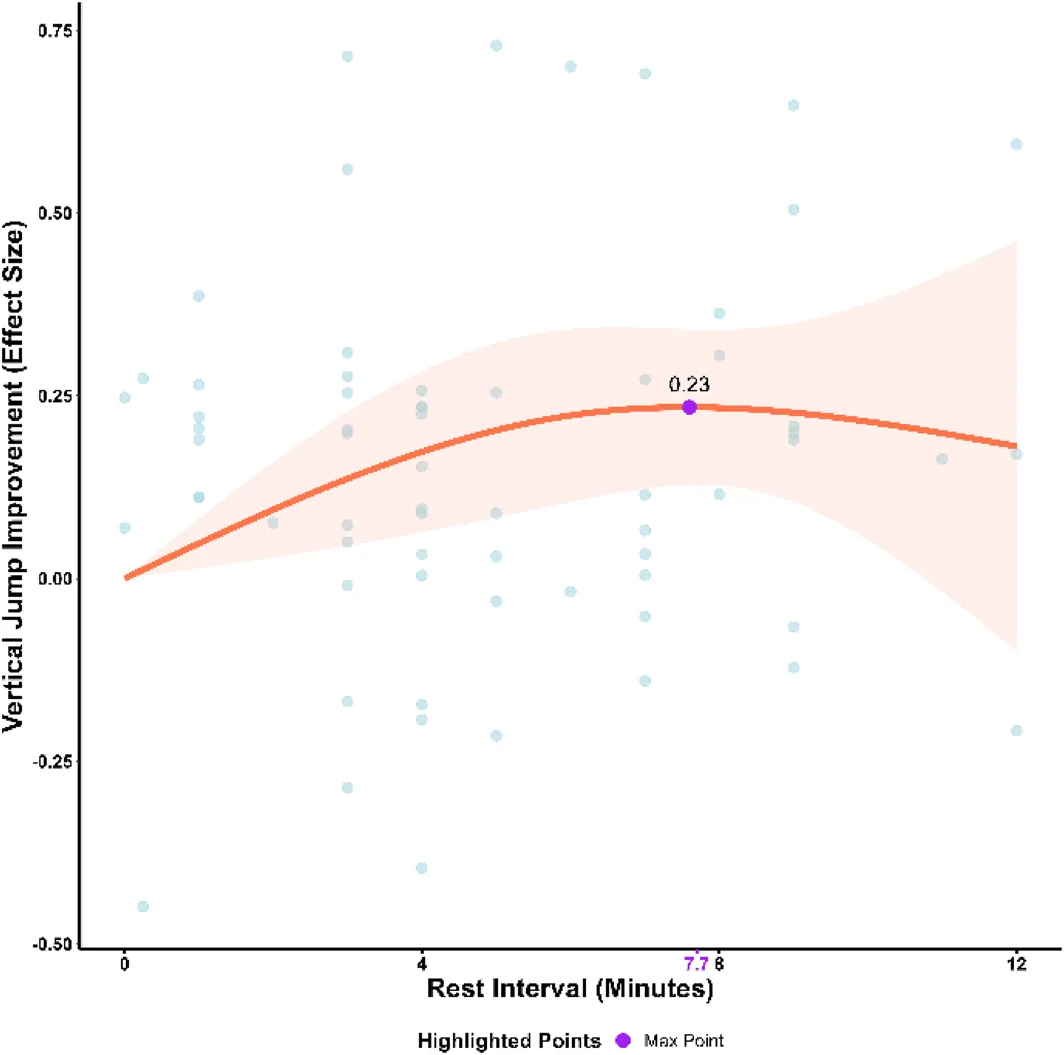
Meta regression of rest interval‐vertical jump height relationship within the isometric group.

**FIGURE 11:**
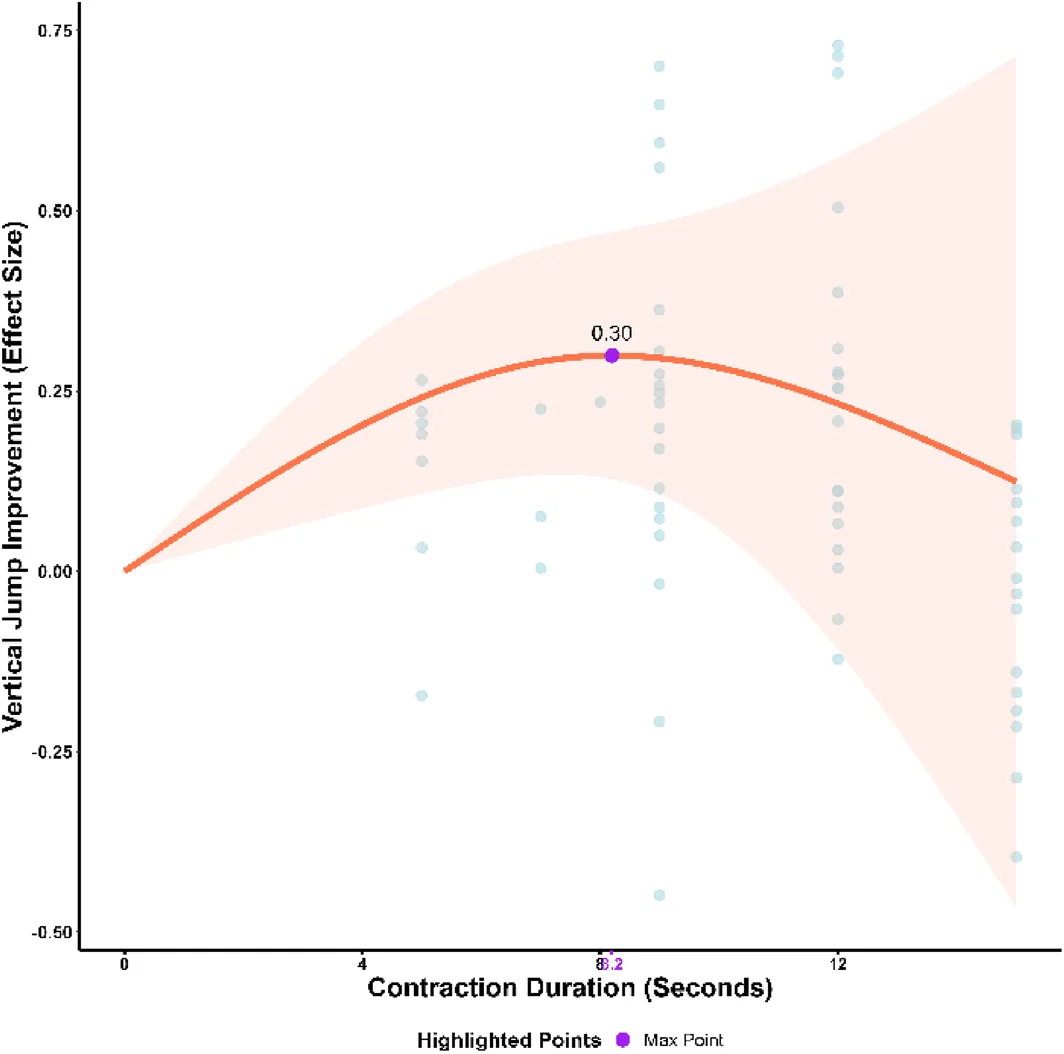
Meta regression of contraction duration‐vertical jump height relationship within the isometric group.

**FIGURE 12:**
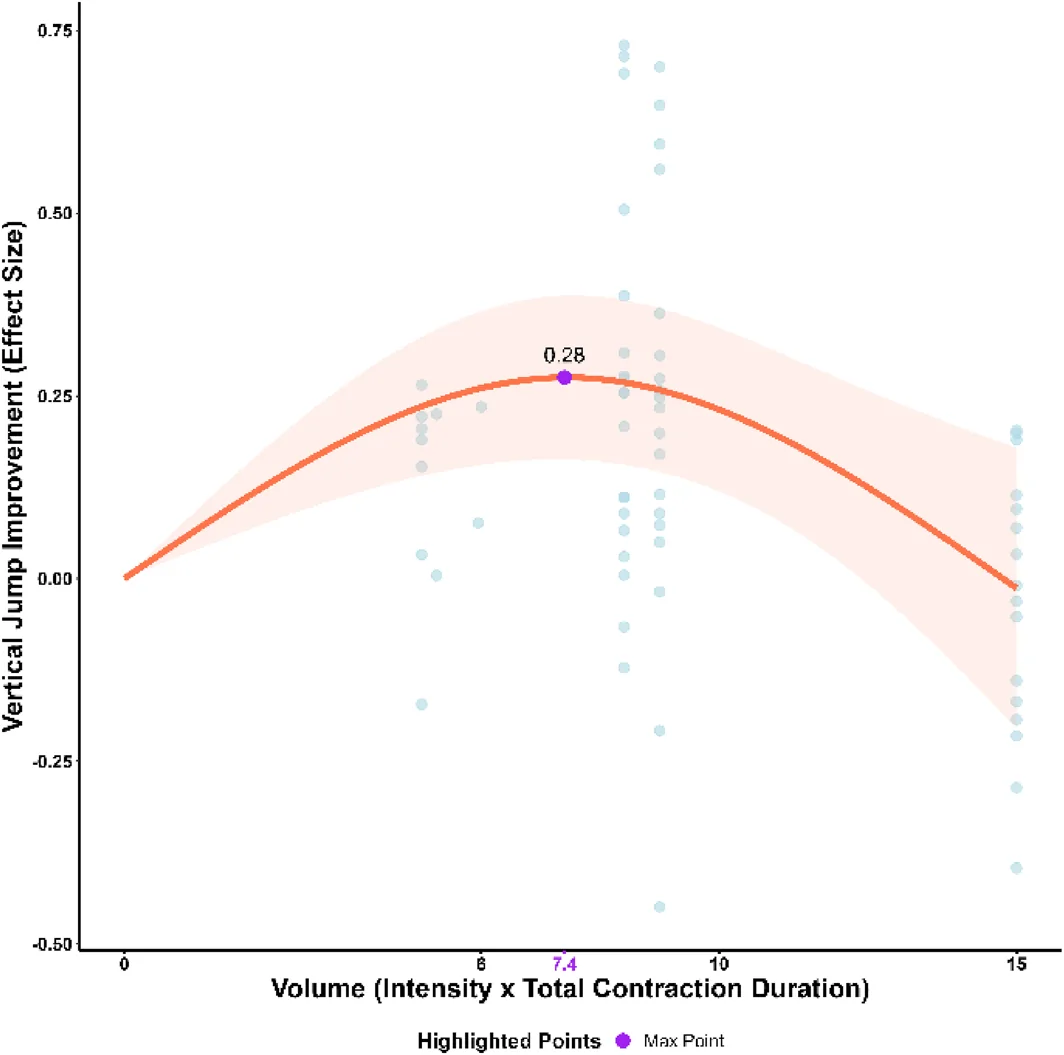
Meta regression of volume‐vertical jump height relationship within the isometric group.

For all three moderators on vertical jump, natural cubic splines provided the best overall fit (Figure 10, 11, 12). For rest interval, the nonlinear model offered only modest gains over a linear trend; we therefore use it illustratively. The curve peaks at around 7.7 min (ES = 0.23), with limited support for nonlinearity and unstable estimates where data are sparse (< 3 or > 10 min). An inverted‐U for the relationship between contraction duration and PAPE emerged with an optimal effect around 8.2 s per session (ES = 0.30). For volume, an inverted‐U emerged with an optimal effect around 7.4 units per session (where 1 unit = 1 s of maximal isometric contraction, or the equivalent intensity‐weighted contraction stimulus) (ES = 0.28). Information‐criterion comparisons favored the same spline specifications across variables (Details in Supporting Information S7 and S9).

## 4 Discussion

This systematic review and meta‐analysis showed that isometric contractions significantly improved vertical jump height, with no clear difference relative to dynamic isotonic contractions. No significant effects were detected for muscular strength or power output. Subgroup‐specific improvements in vertical jump height were observed across several moderator levels, including 8–12 s contraction duration, 6–10 volume units, 4–8 min recovery, multiple repetitions, knee angles of 90°–140°, and in participants with ≥ 2 years of strength training experience. These findings provide useful insights into isometric‐induced PAPE across muscular strength and power outcomes, inform future training guidance for athletes and practitioners, and highlight the need for further work to clarify the mechanisms potentially underlying these responses.

### 4.1 The Overall Effect on Muscular Strength and Power Performance

Our findings suggested that isometric contraction elicited the significant PAPE effect on vertical jump height, whereas no clear differences were detected relative to dynamic isotonic contraction, although the available comparative evidence remained limited. This result contrasts with a prior meta‐analysis (Seitz and Haff 2016), which reported negative effects of ISO on subsequent explosive performance. One possible explanation for this discrepancy lies in the methodological limitations of that study, which included only 14 data points without specifying the number of included primary studies, thereby limiting statistical power and generalizability.

One plausible contributor to the observed PAPE is classic PAP. Although PAP should not be assumed to be the primary underlying mechanism, it may have contributed to performance enhancement under some conditions, particularly at shorter recovery intervals (Sale 2002; Newton et al. 1994; Gossen and Sale 2000). This interpretation is broadly consistent with the observation that significant performance enhancement was also detected within shorter recovery intervals in the present review. Previous studies have shown that PAP can increase the rate of force development and shortening velocity at a given force (or alternatively, increase force production at a given velocity) for 3–4 min following maximal or submaximal isometric contraction (Blazevich and Babault 2019; Stone et al. 2008; DeRenne and Journal 2010; Ebben WPJJoss, 2002). Other physiological factors may also contribute. For example, muscle temperature typically peaks several minutes after high‐intensity exercise and is known to increase RFD and contraction velocity (Blazevich and Babault 2019; Saltin et al. 1968). Increased blood flow and muscle water content following ISO may enhance Ca^2+^ sensitivity, thereby augmenting muscle fiber force and velocity (Blazevich and Babault 2019; Edman and Andersson 1968). However, because none of these mechanisms were directly measured in the included studies, these interpretations should be considered provisional and hypothesis‐generating rather than definitive.

Our meta‐analysis did not show significant effects of ISO on muscular strength or power output. These null findings should be interpreted cautiously because the number of available studies was small and the assessment methods were heterogeneous. One possible interpretation is that PAP‐related mechanisms may be more relevant to explosive or submaximal force‐velocity tasks than to maximal force production. Current evidence suggests that PAP does not typically increase maximal force (Tillin and Bishop 2009) but may enhance the RFD during MVC, movement velocity at a given load (Sale 2002; Gossen and Sale 2000), and submaximal force for a given level of neural activation during brief explosive activities (i.e., jump, sprint, and stroke cycle) (Blazevich and Babault 2019; Gossen and Sale 2000). A related explanation is that MRLC phosphorylation, a key mechanism underpinning PAP, is maximized at low levels of calcium (i.e., low‐frequency tetanic contractions) (Sweeney and Stull 1990), whereas tasks requiring maximal or near‐maximal levels of muscle activation (i.e., high‐frequency tetanic contractions) may not directly benefit from it (Blazevich and Babault 2019; Vandenboom et al. 1993; Abbate et al. 2000). Although PAPE may also involve other physiological processes (e.g., increased muscle temperature, fluid shifts, or motor unit recruitment) (Fink et al. 1986), the current evidence does not allow firm mechanistic conclusions regarding the absence of a significant strength or power output effect. The null findings of power output may partly reflect methodological limitations, as some studies relied on indirect estimates rather than direct laboratory‐based measurements (Sayers et al. 1999). For example, one study (Rixon et al. 2007) reported significant improvements in CMJ height and peak power after isometric contraction, but peak power was estimated from CMJ height rather than directly measured using a force platform, which is typically considered the standard method for assessing actual power output (Sayers et al. 1999). These limitations highlight the need for more well‐controlled studies using valid and direct assessments of power outcomes to clarify the PAPE effects of ISO.

### 4.2 Factors Modulating PAPE of Isometric Contraction

#### 4.2.1 Rest Interval

Based on the moderator analysis and meta‐regression, rest interval appeared to show a possible inverted U‐shaped relationship with vertical jump height. Moderator analysis suggested that vertical jump height was significantly enhanced within 0–8 min after ISO, with the greatest improvement generally observed at 4–8 min, whereas the effect appeared to attenuate beyond 8 min. However, this temporal pattern should be interpreted cautiously because the certainty of evidence was low and some interval categories, particularly those beyond 8 min, were informed by few studies.

Different mechanisms may contribute across recovery windows. One possible explanation relates to the energetic characteristics of the ISO protocols, which primarily relied on the phosphagen system to support maximal‐effort activities lasting up to 15 s (Gastin 2001). Phosphagen restoration is generally rapid and may be largely re‐established within several minutes of rest (Ellington 2001), which is broadly consistent with the performance enhancement observed in shorter recovery windows, although the available data do not directly support phosphagen recovery as the explanation for the reduced effect at longer intervals. The enhancement observed at 4–8 min may also reflect other mechanisms, such as elevated muscle temperature, which has been reported to peak around 5 min after high‐intensity contractions (Blazevich and Babault 2019). Interestingly, this pattern differs from that reported in studies using dynamic isotonic contractions, which often show impaired vertical jump performance within 0–3 min post‐exercise and improvement only after 8–12 min of recovery (Gouvêa et al. 2013). Although the relatively broad 0–4 min effective window observed here may partly reflect the limited number of studies, some evidence suggests that significant PAPE can emerge even after very short rest intervals (Downey et al. 2022; French et al. 2003). This may be related to contraction mode‐specific fatigue responses: isometric exercise tends to induce greater central but less peripheral fatigue, whereas the opposite has been reported for dynamic contractions (Babault et al. 2006; Karelis et al. 2004). As central fatigue usually recovers more rapidly (within ∼2 min), whereas peripheral fatigue may persist for 3–5 min or longer after high‐intensity exercise (Carroll et al. 2017), the earlier emergence of performance enhancement after ISO protocols may partly reflect faster recovery from central fatigue (Xu et al. 2024; Seitz and Haff 2016). However, whether this fully explains the earlier onset of performance enhancement after ISO protocols remains uncertain, and the timing of ISO‐induced PAPE may also be influenced by multiple factors.

##### 4.2.1.1 Intensity and Volume

Regarding intensity and volume, moderator analyses suggested some subgroup‐specific effects, whereas the between‐level differences were not consistently robust. Accordingly, current evidence does not support a definitive conclusion that intensity or volume independently determines the magnitude of the PAPE response. For intensity, subgroup‐specific patterns suggested that higher‐intensity protocols were not always associated with more favorable vertical jump responses, possibly because they imposed greater acute fatigue. However, this interpretation is confounded by protocol characteristics: Submaximal intensities were almost exclusively implemented using HIMA, whereas maximal intensities were predominantly implemented using PIMA. Because HIMA and PIMA differ in motor control demands and motor unit recruitment strategies, they may induce different PAPE responses (Oranchuk et al. 2024). Interpretation is further complicated by differences in the prescription method, as most submaximal protocols prescribed intensity based on dynamic strength (i.e., RM), whereas maximal protocols used MVC. Thus, the apparent effect of intensity may partly reflect isometric modality and prescription method rather than intensity per se.

In this review, volume was defined as an intensity‐weighted contraction stimulus, and more favorable effects were observed with moderate contraction duration (8–12 s), multiple repetitions, and moderate volume (6–10 units) than with shorter or longer durations, single repetitions, and lower or higher volumes. These patterns may reflect the balance between fatigue and potentiation, as PAPE may be more likely to emerge when fatigue is adequately managed (Tillin and Bishop 2009). Although high‐volume protocols may increase peripheral fatigue through reductions in phosphocreatine and glycogen, metabolite accumulation, and muscle damage (Walker et al. 2012; D. G. Allen et al. 2008), substantial phosphocreatine depletion and lactate accumulation generally require contractions of ∼37 s or longer (MacDougall et al. 1999), whereas most long‐duration protocols in this review lasted < 15 s. A plausible explanation is that moderate durations provide sufficient neural and mechanical stimulus to induce potentiation without excessive fatigue. Consistent with this interpretation, Vandervoort et al. 1983 reported the greatest twitch potentiation after a 10‐s maximal isometric contraction, whereas very short durations or low volumes may provide insufficient stimulus to induce an effective PAPE response. Research found that the motor unit recruitment and rate coding for ISO (especially submaximal HIMA) were likely regulated by the size principle, which indicated that more and higher order motor units can be recruited and activated as the muscle approaching a certain level of fatigue (Henneman et al. 1965).

#### 4.2.2 Joint Angle

In our study, lower‐limb ISO protocols using knee angles of 90°–140° were associated with larger improvements in vertical jump height than protocols using knee angles ≤ 90°. Current literature emphasizes the importance of kinematic similarity between the CA and subsequent performance task, suggesting that the joint angle during ISO may be an important determinant of the PAPE response (Blazevich and Babault 2019). However, because most included studies allowed participants to self‐select jump depth, the exact knee angles used during jump testing could not be determined, limiting direct comparison between the conditioning and performance tasks. Although Bobbert et al. 1996 reported an average knee angle of approximately 75° during self‐selected countermovement jumps, suggesting that jump knee angles typically fall below 90°, differences in joint angle alone may not fully explain the present findings. A possible explanation is that ISO performed at smaller knee angles (i.e., longer muscle lengths) may induce greater blood flow restriction, oxygen consumption, and metabolite accumulation than contractions performed at larger angles (Oranchuk et al. 2024; de Ruiter et al. 2005), thereby increasing neuromuscular fatigue and attenuating the subsequent PAPE response. However, this interpretation remains speculative. Overall, the available evidence suggests that the joint angle during ISO may influence the magnitude of the PAPE effect, and future studies should clarify how different joint angles acutely affect muscle architecture, neuromuscular function, and subsequent performance.

#### 4.2.3 Training Modality

Regarding training modalities, both HIMA and PIMA significantly enhanced vertical jump height, although HIMA showed a larger subgroup‐specific effect. Oranchuk et al. 2024 highlighted that HIMA and PIMA involve distinct neuromuscular control strategies, which may lead to different neural, musculoskeletal, and cardiovascular stimuli. Suchomel et al. 2016 further proposed that reflex potentiation, as a component of PAP, may contribute to explosive tasks such as vertical jumping, and its magnitude may vary according to motor unit recruitment, discharge behavior, and movement specificity (Duclay and Martin 2005; Baudry et al. 1985. 2005; Ritzmann et al. 2013; Gruber et al. 2007; Cochrane 2011). Previous studies have shown that HIMA is associated with greater central demands and enhanced reflex responsiveness, which may potentially explain its larger effect. In addition, lengthening contractions, which are more characteristic of HIMA, are associated with lower peripheral but greater central neural activity (Fang et al. 2001; Perrey 2018). Because central fatigue typically dissipates faster than peripheral fatigue (Carroll et al. 2017), this may facilitate an earlier recovery of explosive performance.

PIMA, which is suggested as a shortening task, may also be considered an effective approach for inducing PAPE, particularly with explosive intent (Schaefer and Bittmann 2017; Oranchuk et al. 2024). Contractions performed with explosive intent may be more favorable for enhancing RFD and muscle power, potentially through the preferential recruitment of higher‐threshold motor units (Oranchuk, Storey, Nelson, and Cronin 2019a; Schaefer and Bittmann 2017; Behm et al. 2025). These acute responses may also contribute to improved subsequent jump performance. Future studies should compare neuromuscular activity during and after HIMA and PIMA, together with performance outcomes assessed across different recovery intervals, to clarify their distinct contributions to the PAPE effect and better inform practice.

#### 4.2.4 Participant Characteristics

Regarding participant's characteristics, our meta‐analysis showed that individuals with ≥ 2 years of strength training experience significantly improved vertical jump height after ISO, whereas those with < 2 years did not. This is broadly consistent with Seitz et al.’ study (Seitz and Haff 2016), who reported greater PAPE responses after dynamic isotonic resistance exercise in stronger individuals, potentially reflecting greater fatigue resistance and other training‐related adaptations (Seitz and Haff 2016; Seitz et al. 2014). Individuals with more strength training experience may also possess a greater proportion and cross‐sectional area of type II muscle fibers (Seitz and Haff 2016; Maughan et al. 1983; Aagaard and Andersen 1998), which may increase responsiveness to mechanisms proposed to underpin PAP/PAPE (Tillin and Bishop 2009). In addition, other PAPE‐related mechanisms may also depend on muscle fiber characteristics. For example, the effects of muscle blood flow and water content on force and shortening velocity appear greater in fast‐twitch than slow‐twitch fibers in mammalian muscle (Fink et al. 1986), which may partly explain the larger PAPE effect observed in more strength‐experienced individuals.

Our analyses suggested a significant PAPE effect in male participants, whereas the corresponding effect in female participants was not statistically significant. However, this finding should be interpreted cautiously. The pooled sample was heavily male‐dominated (601 men vs. 181 women), making the female estimate more vulnerable to imprecision and small‐study effects. In addition, the female participants generally displayed lower relative strength levels (mostly 1.0–1.3 × bodyweight squat) than the male participants (1.2–1.83 × bodyweight), so the apparent sex‐related difference may have been partly confounded by the strength level rather than sex per se. Potential physiological differences may also be relevant. Previous studies suggest that males and females differ in body composition and muscle architecture (Miller et al. 1993; Bartolomei et al. 2021), and males may be stronger than females despite similar training experience (Bishop et al. 1987). Evidence regarding sex differences in fatigue recovery remains inconclusive: females have been reported to be more fatigue‐resistant than males in intermittent handgrip isometric tasks (Hunter et al. 2009), whereas only minor sex differences in central and peripheral fatigue were observed after isometric plantar flexion to task failure (Jo et al. 2022). They suggested a greater role of central fatigue in males compared with females, while more peripheral fatigue in females (Jo et al. 2022). At present, the extent to which these differences influence PAPE remains unclear. Therefore, more studies with adequately represented female samples are needed before firm conclusions can be drawn regarding sex differences in isometric‐induced PAPE. Future studies should also match participants by relative strength or account for strength statistically, and report menstrual cycle status and hormonal contraceptive use to improve the interpretation of female‐specific responses.

### 4.3 Limitation

Several limitations should be considered when interpreting the present findings. First, most participants were strength‐trained, which may limit generalizability to untrained or recreationally active populations. In addition, the current literature has focused mainly on lower‐limb isometric contraction; therefore, the extent to which these findings generalize to upper‐limb or whole‐body tasks remains unclear. Second, the overall certainty of evidence was limited, and risk of bias was rated as high or unclear across several domains, despite generally adequate reporting of interventions and outcome measures. This pattern appeared to reflect, at least in part, methodological constraints that are common in acute exercise‐based interventions—such as the practical difficulty of blinding—as well as uncertainty in several PAPE‐specific domains, rather than uniformly poor study design. Although the review protocol was registered before data extraction and no changes were made thereafter, registration occurred after the literature search and study screening had been completed and should therefore be regarded as a transparency measure rather than a fully prospective registration. Third, for muscular strength and power outcomes, as well as comparisons between ISO and DYN, the number of studies was small, assessment methods were heterogeneous, and some findings were sensitive to influential effect sizes; therefore, these conclusions should be considered more tentative. Finally, moderator findings should be interpreted cautiously. A relatively large number of moderator analyses were conducted, which increases the possibility of multiplicity‐related findings. In addition, some moderator levels were informed by few studies or effect sizes, and several moderators were conceptually related. Intensity‐related findings were difficult to interpret independently because submaximal and maximal protocols also differed in training modality and prescription method. Potential interactions between moderators were not examined, and multivariable meta‐regression was not pursued because of the limited number of studies. Accordingly, moderator findings, particularly sex‐related findings derived from a male‐dominated sample, should be viewed as exploratory rather than definitive.

## 5 Conclusion

This systematic review and meta‐analysis suggested that isometric contraction can be an effective strategy for eliciting PAPE in vertical jump height. A rest interval of 0–8 min appeared sufficient to produce significant improvements, with larger effects generally observed within 4–8 min post‐intervention, although evidence for longer intervals was limited. No clear differences were detected between isometric contraction and dynamic isotonic contractions. Several participant‐ and protocol‐related characteristics were associated with subgroup‐specific patterns in vertical jump PAPE. No significant effects were detected for muscular strength or power output. Future research should further examine the participant‐ and protocol‐related factors associated with PAPE responses to isometric contraction across diverse populations to enable more targeted and effective programming in applied contexts.

## 6 Practical Applications

**FIGURE 13:**
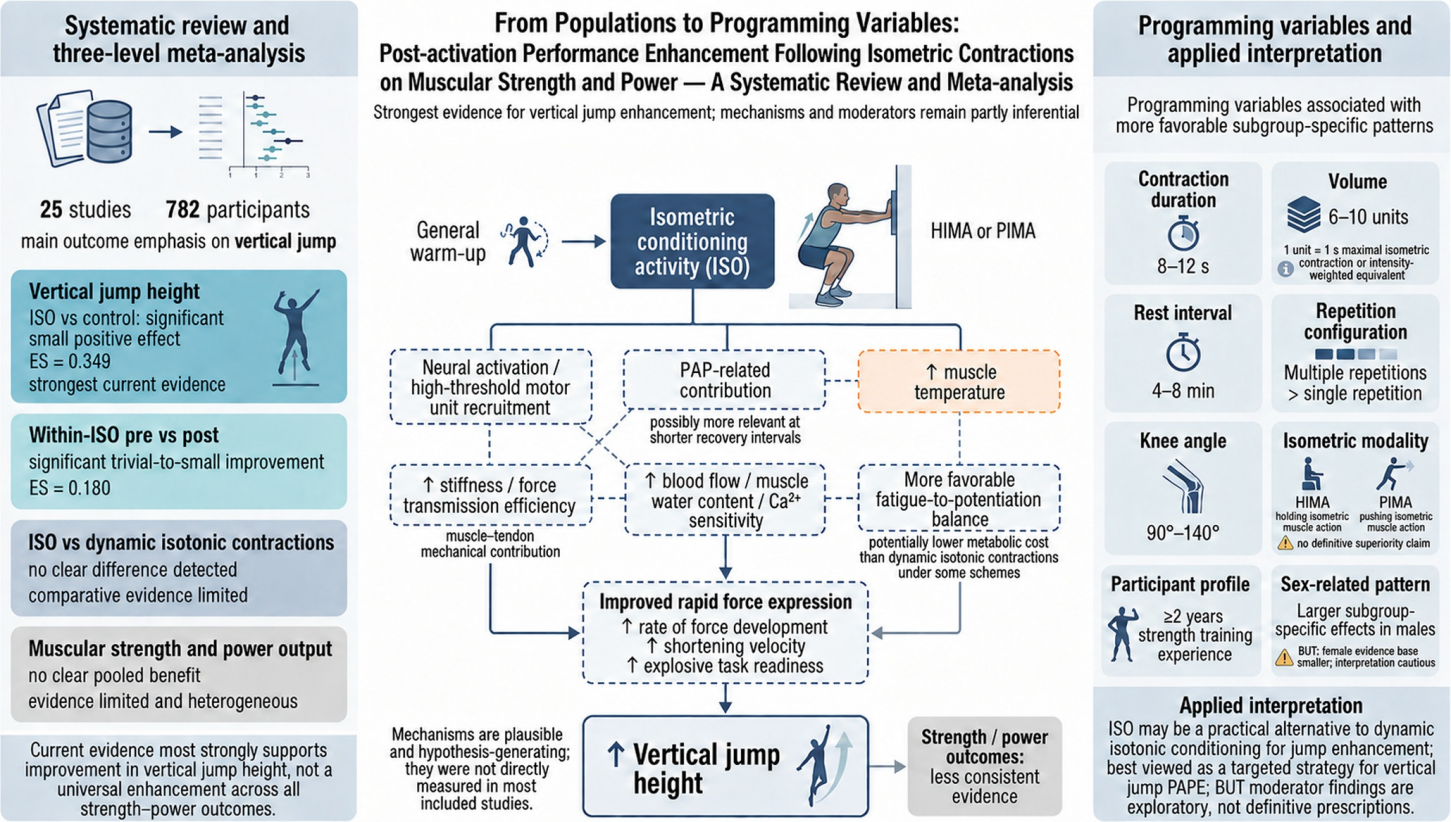
The graphical abstract and practical applications.

Compared with control conditions, ISO was associated with greater acute improvements in vertical jump height. More favorable patterns were generally observed with 8–12 s contraction duration, 6–10 volume units, 4–8 min recovery, multiple repetitions, knee angles of 90°–140°, and in participants with ≥ 2 years of strength training experience. Both HIMA and PIMA appeared potentially useful in practice, but current evidence is insufficient to make definitive superiority claims (Figure 13).

## Author Contributions

Zhijian He conceived the idea for the article. Zhijian He, Zhipeng Xue and Lijun Qiang performed the literature search, data extraction and data analysis. Zhijian He, Zhipeng Xue and Lijun Qiang wrote the first draft. The revisions were made by Dapeng Bao, Junhong Zhou, and Ruinan Zhang with critical input from Ji Tu, Qiushi Wang and Yilin Xu. All the figures and tables were made by Zhijian He, Zhipeng Xue and Zepeng Lu. All authors read and approved the final manuscript.

## Funding

The authors have nothing to report.

## Ethics Statement

The authors have nothing to report.

## Consent

The authors have nothing to report.

## Conflicts of Interest

The authors declare no conflicts of interest.

## Supporting information


Supporting Information S1

Supporting Information S2

Supporting Information S3

Supporting Information S4

Supporting Information S5

Supporting Information S6

Supporting Information S7

Supporting Information S8

Supporting Information S9

Supporting Information S10

## Data Availability

The data that support the findings of this study are available from the corresponding author upon reasonable request.
