# Supplementary material for: From Populations to Programming Variables: Post‐Activation Performance Enhancement Following Isometric Contractions on Muscular Strength and Power—A Systematic Review and Meta‐Analysis
Source: Eur J Sport Sci. 2026 May 19;26(6):e70183. doi: 10.1002/ejsc.70183 (PMC13580768; doi:10.1002/ejsc.70183)
Supplement: Supplementary file 1 [file EJSC-26-e70183-s001.docx]

**Meta-regression model results:**

1. **Meta regression on the relationship between rest interval and vertical jump**

**Information-criterion comparisons**

| Rank | Model | LogLik | Deviance | AIC | BIC | AICc | ΔAIC | ΔAICc | ΔBIC |
| --- | --- | --- | --- | --- | --- | --- | --- | --- | --- |
| 1 | Linear | -4.155566 | 4.382871 | 12.31113 | 16.69044 | 12.50161 | 0.00000 | 0.00000 | 0.00000 |
| 2 | Natural Cubic Spline | -3.451744 | 4.290383 | 12.90349 | 19.47245 | 13.29058 | 0.59236 | 0.78897 | 2.78201 |
| 3 | Restricted Cubic Spline | -8.715311 | 17.430623 | 21.43062 | 25.74839 | 21.62110 | 9.11949 | 9.11949 | 9.05795 |
| 4 | Thin Plate Spline | -12.653699 | 5.670193 | 29.30740 | 33.68671 | 29.49787 | 16.99627 | 16.99626 | 16.99627 |
| 5 | Cubic Polynomial | -12.575682 | 5.656803 | 33.15136 | 41.90998 | 33.80710 | 20.84023 | 21.30549 | 25.21954 |

**2. Meta regression on the relationship between contraction duration and vertical jump**

**Information-criterion comparisons**

| Rank | Model | LogLik | Deviance | AIC | BIC | AICc | ΔAIC | ΔAICc | ΔBIC |
| --- | --- | --- | --- | --- | --- | --- | --- | --- | --- |
| 1 | Natural Cubic Spline | 3.212849 | 3.447514 | -0.42570 | 6.09746 | -0.03226 | 0.00000 | 0.00000 | 0.00000 |
| 2 | Restricted Cubic Spline | -5.522502 | 11.045004 | 15.04500 | 19.33127 | 15.23855 | 15.47070 | 15.27081 | 13.23381 |
| 3 | Linear | -6.021487 | 4.580402 | 16.04297 | 20.39175 | 16.23652 | 16.46867 | 16.26878 | 14.29428 |
| 4 | Thin Plate Spline | -8.105063 | 4.883669 | 22.65960 | 29.67142 | 23.10792 | 23.08529 | 23.14018 | 23.57396 |
| 5 | Cubic Polynomial | -7.643093 | 4.814741 | 23.28619 | 31.98374 | 23.95285 | 23.71188 | 23.98511 | 25.88627 |

**3.Meta-regression on the relationship between volume and vertical jump**

**Information-criterion comparisons**

| Rank | Model | LogLik | Deviance | AIC | BIC | AICc | ΔAIC | ΔAICc | ΔBIC |
| --- | --- | --- | --- | --- | --- | --- | --- | --- | --- |
| 1 | Natural Cubic Spline | 2.881684 | 3.482822 | 0.23663 | 6.75979 | 0.63007 | 0.00000 | 0.00000 | 0.00000 |
| 2 | Restricted Cubic Spline | -4.979221 | 9.958442 | 13.95844 | 18.24471 | 14.15199 | 13.72181 | 13.52192 | 11.48492 |
| 3 | Linear | -7.583373 | 4.805902 | 19.16675 | 23.51552 | 19.36029 | 18.93011 | 18.73022 | 16.75573 |
| 4 | Thin Plate Spline | -7.892598 | 4.851847 | 21.38448 | 27.47198 | 21.73211 | 21.14785 | 21.10204 | 20.71219 |
| 5 | Cubic Polynomial | -7.721400 | 4.826356 | 23.44280 | 32.14035 | 24.10947 | 23.20617 | 23.47939 | 25.38056 |
