# Supplementary material for: From Populations to Programming Variables: Post‐Activation Performance Enhancement Following Isometric Contractions on Muscular Strength and Power—A Systematic Review and Meta‐Analysis
Source: Eur J Sport Sci. 2026 May 19;26(6):e70183. doi: 10.1002/ejsc.70183 (PMC13580768; doi:10.1002/ejsc.70183)
Supplement: Supplementary file 2 [file EJSC-26-e70183-s002.docx]

**Supplementary material on methods and results**

1. **Methods**
   1.1 **Moderator Analysis and Meta-regression**

To investigate the effects of variables potentially contributing to the PAPE of isometric contraction, moderators that was conceptually associated with the outcomes and each with at least 8 effect sizes were included in the meta-analysis [52]. Some moderator levels were excluded due to insufficient data or ambiguous reporting (e.g., mixed-sex groups, unclear joint angles). For vertical jump, the moderators were: sex (male and female), strength training experience (< 2 years and ≥ 2 years), intensity (maximal and submaximal), contraction duration (short, moderate, long), volume (low, moderate, high), rep configuration (single rep and multi reps), intervention modality (HIMA and PIMA), joint angle (knee angle ≤ 90°, 90°＜knee angle ≤ 140°), and rest interval (short, moderate, long). For within isometric group comparison of strength performance, the moderators included strength training experience (strength untrained and strength trained), intensity (maximal and submaximal), contraction duration (short, moderate, long and prolonged), volume (low, moderate, high and very high), rep configuration (single rep and multi reps), and rest interval (short, moderate, long and prolonged). No moderators were analyzed for between-group comparisons (isometric vs. concentric) in strength outcomes. For power output outcomes, the moderators were sex, strength training experience, volume, contraction duration, and rest interval. Moderator models with and without intercepts were analyzed to assess the effects of each level relative to the reference category. Moderator effects were estimated along with 95% confidence intervals (CIs) and, where applicable, 95% prediction intervals (PIs). To further characterize the relative contributions of the identified moderators, multiple linear regression was performed within the isometric group using IBM SPSS Statistics software. Standardized beta coefficients (Std β) were computed, and variance inflation factors (VIFs) were used to assess potential collinearity. VIFs below 2.5 indicated no significant multicollinearity.

In order to explore the relationship between different continuous variables (contraction duration, volume and rest interval) and isometric-induced PAPE, the linear and non-linear meta-regressions were conducted within isometric contraction group (pre vs post comparison). All models were evaluated using both linear and various nonlinear meta-regression models, including Simple Linear, Cubic Polynomial, Restricted Cubic Spline, Natural Cubic Spline, and Thin Plate Spline Models [53]. The optimal fitting model was selected based on goodness-of-fit metrics and practical considerations [54]. Considering the nature of PAPE effect, which are likely to follow an inverted U-shaped pattern [9, 11], although the linear model exhibited a better fit indices (e.g., lower Akaike Information Criterion and deviance), its predictive performance was deemed implausible from a practical standpoint. Consequently, the nonlinear model with the best goodness-of-fit was selected for the final analysis. Additionally, for nonlinear models demonstrating a clear inverted U trend, we extracted the peak points (the maximum effect size within the fitted profile) partially adopted from the methodology outlined by Xu et al [9]. Among all nonlinear models assessed in this study, the Natural Cubic Spline provided the best goodness-of-fit. All regression analyses were conducted using the metafor package and subsequently visualized with the ggplot2 package in R.

2. **Results**
   2.1 **Study Selection and Study Characteristics**

A total of 5,958 studies were retrieved through database screening: PubMed (n=921), EBSCO (n=943), Web of Science (n=1,303), and Scopus (n=2,791). After removing 2,371 duplicates using EndNote software, 3,587 studies were excluded based on their titles and abstracts. A total of 38 studies were deemed eligible for further evaluation. Following full-text screening, 25 studies were included in the final meta-analysis, with 22 identified through database searches and 3 through other methods [24-30, 32, 34, 36, 47, 61-74].

The 25 studies included in this meta-analysis involved a total of 782 participants (601 males and 181 females) aged 17 to 52 years (mean age: 23.45 ± 5.11 years). Nineteen included strength-trained individuals, 4 of untrained individuals, and 2 included both strength-trained and untrained participants. In terms of strength training experience, 10 studies reported participants of < 2 years, while 14 studies of ≥ 2 years, with one study encompassing participants of both groups. Regarding the isometric contraction protocols, 24 studies primarily targeted lower-limb muscles (2 studies used isometric leg press, 14 focused on isometric squats, 4 on isometric knee extensions, 1 on multiple isolated lower-limb muscle contractions, 1 on plantar flexion, and 2 on isometric hip-hinge exercises), while 3 studies targeted upper-limb muscles (all utilizing isometric bench press). The intensity of the isometric contraction ranged from 20% to 100% of maximal voluntary contraction. Among these, 18 studies employed maximal intensity, and 8 studies used submaximal intensity (2 studies at < 60% maximal intensity and 6 studies at 70-85% maximal intensity). The total contraction duration across studies ranged from 5 to 60 seconds. Specifically, 8 studies employed “short duration”, 8 studies of “moderate duration”, 13 studies of “long duration”, and 1 study of “prolonged duration”. The volume varied from 5 to 60 units, with 8 studies classified as “low volume,” 13 studies as “moderate volume,” 8 studies as “high volume,” and 1 study as “very high volume.” Regarding repetition configurations, 8 studies utilized a “single repetition” protocol, while 19 studies employed “multiple repetitions.” Addition-ally, 18 studies used “PIMA” and 6 studies used “HIMA.” Concerning the angle of knee flexion during lower-limb isometric contraction, the reported ranged was 60-140°. Specifically, 15 studies reported a knee angle ≤90°, and 4 studies reported a knee angle between 90° and 140°. As for the outcome measurement, 20 studies assessed vertical jump performance (17 studies focused on CMJ, 3 on SJ, and 2 on DJ), 7 studies reported strength outcomes, and 4 measured power output. The rest interval varied from 0 to 60 minutes post-intervention: 15 studies reported “short interval”, 17 studies of “moderate interval”, 11 studies of “long interval”, and 1 study measured at a “prolonged interval”.

2.2 **Risk of Bias Results**

All studies included in this review reported randomization; however, only eight studies [1-8] provided specific details regarding their randomization procedures. These studies were classified as “low risk”, while the remaining studies were rated as “unclear risk”. None of the studies employed blinding of testers. Attrition was observed in seven studies [3-9], leading to an “unclear risk” of attrition bias, while the other studies were classified as “low risk”. Three studies [10-12] conducted more than two familiarization trials, resulting in a “low risk” classification for familiarization, whereas 16 studies [1, 3-6, 8, 9, 13-21]performed only a single familiarization trial, and six studies [2, 7, 22-25] omitted a familiarization phase altogether. 13 studies [2, 8, 10, 11, 13-16, 18, 22-25]assessed a single time point and were rated as “low risk”. In contrast, 12 studies [1, 3-7, 9, 12, 17, 19-21]measured multiple time points with intervals of ≥4 minutes, which were classified as “unclear risk”. 11 studies [3, 5, 8, 10, 11, 15, 16, 19, 23-25] did not report reliability statistics, eight studies [2, 9, 12-14, 17, 18, 22]reported only one statistic, and six studies [1, 4, 6, 7, 20, 21], which provided multiple reliability indicators, were classified as “low risk”. Twelve studies [3-10, 16, 17, 20, 22]conducted a priori sample size estimations and were rated as “low risk”, while 13 studies [1, 2, 11-15, 18, 19, 21, 23-25], which did not perform a priori sample size calculations but had a sample size of ≥10 participants, were classified as “unclear risk”.

2.3 **Meta-analysis Results**

**2.3.1 Main Effect**

Compared to the control group, a significantly greater effect on vertical jump height in the isometric group was observed with a moderate effect size (ES = 0.406, SE = 0.127, t = 3.204, df = 52, p = 0.002, 95% CI [0.152, 0.660], 95% PI [−0.560, 1.372]). Substantial heterogeneity was detected across studies (Q (df = 52) = 125.187, p < 0.0001), with a total I² value of 60.65% (I²-level 2 = 18.64%, I²-level 3 = 42.01%), indicating that a significant portion of variance in effect sizes was due to between-study differences. After excluding four influential effect sizes [3, 12, 22, 23] identified through sensitivity analysis, the revised model remained significant, with a slightly smaller effect size (ES = 0.349, SE = 0.110, t = 3.175, df = 48, p = 0.003, 95% CI [0.128, 0.569], 95% PI [−0.365, 1.062], I²-level 2 = 7.46%, I²-level 3 = 37.58%). The revised model was retained due to the narrower confidence interval providing a more robust estimation**.** However, no significant difference in vertical jump height between the isometric contraction and the dynamic isotonic contraction was observed (ES = −0.061, SE = 0.126, t = −0.483, df = 8, p = 0.642, 95% CI [−0.352, 0.230], 95% PI [−0.352, 0.230]) **(Figure 7)**. No heterogeneity was detected across studies (Q (df = 8) = 1.002, p = 0.998), with a total I² value of 0%. Sensitivity analysis identified no influential studies.

A significant improvement in vertical jump height was observed within the isometric contraction group with a small effect size (ES = 0.157, SE = 0.054, t = 2.912, df = 69, p = 0.005, 95% CI [0.049, 0.264], 95% PI [−0.114, 0.427]). No significant heterogeneity was detected across studies (Q (df = 69) = 47.215, p = 0.979), and the total I² value was 11.65% (I²-level 2 = 0%, I²-level 3 = 11.65%), indicating a low heterogeneity. After excluding four influential effect sizes [2, 12, 23] identified through sensitivity analysis, the revised model showed a significant effect with a slightly larger effect size (ES = 0.180, SE = 0.051, t = 3.518, df = 65, p = 0.0008, 95% CI [0.078, 0.282], 95% PI [−0.041, 0.401], I²-level 2 = 0%, I²-level 3 = 7.67%). The revised model was retained due to the narrower confidence interval providing a more robust estimation.

No between group comparison analysis on power output was performed due to the limited number of studies. No significant effect on power output was observed within the isometric contraction group (ES = −0.130, SE = 0.315, t = −0.412, df = 26, p = 0.684, 95% CI [−0.777, 0.517], 95% PI [−1.549, 1.289]). Substantial heterogeneity was observed (Q (df = 26) = 74.100, p < 0.0001, I²-level 2 = 11.34%, I²-level 3 = 56.59%). After excluding four influential effect sizes [14, 21, 25] identified through sensitivity analysis, the revised model yielded a similarly non-significant result (ES = −0.371, SE = 0.424, t = −0.875, df = 22, p = 0.391, 95% CI [−1.249, 0.508], 95% PI [−1.871, 1.130], I²-level 2 = 0.2%, I²-level 3 = 65.57%). Because the exclusion of influential effect sizes was conducted as a sensitivity analysis rather than based on prespecified reasons for removal, the original model including all eligible effect sizes was retained as the primary analysis. Nevertheless, the limited evidence base and substantial heterogeneity warrant cautious interpretation.

No significant difference in muscular strength performance between the isometric contraction and the control groups was observed (ES = 0.542, SE = 0.336, t = 1.615, df = 9, p = 0.141, 95% CI [−0.217, 1.301], 95% PI [−1.010, 2.095]). Substantial heterogeneity was detected (Q (df = 9) = 29.426, p < 0.001), with a total I² value of 72.06% (I²-level 2 = 0%, I²-level 3 = 72.06%), indicating that most of the variance in effect sizes was attributable to between-study differences. After excluding one influential effect size [14] identified through sensitivity analysis, the revised model yielded no significant difference (ES = 0.657, SE = 0.408, t = 1.612, df = 8, p = 0.146, 95% CI [−0.283, 1.597], 95% PI [−1.127, 2.441], I²-level 2 = 0%, I²-level 3 = 76.31%). The original model was retained to incorporate all available studies, providing a broader perspective on the overall effect size. Nevertheless, the limited number of studies and substantial between-study heterogeneity warrant cautious interpretation (Figure 8).

No significant effect on muscular strength was observed within the isometric contraction group (ES = 0.233, SE = 0.309, t = 0.754, df = 57, p = 0.454, 95% CI [−0.386, 0.852], 95% PI [−2.082, 2.548]). Substantial heterogeneity was detected (Q (df = 57) = 336.030, p < 0.0001), with a total I² value of 87.73% (I²-level 2 = 68.81%, I²-level 3 = 18.92%). After excluding nine influential effect sizes [10, 11, 14, 16, 19, 23] identified through sensitivity analysis, the revised model yielded no significant effect (ES = 0.191, SE = 0.367, t = 0.520, df = 48, p = 0.606, 95% CI [−0.547, 0.928], 95% PI [−1.874, 2.255], I²-level 2 = 61.00%, I²-level 3 = 22.93%). Because the exclusion of influential effect sizes was conducted as a sensitivity analysis rather than based on prespecified reasons for removal, and because the number of studies contributing to the strength outcome was already limited, the original model including all eligible effect sizes was retained as the primary analysis. Nevertheless, the very high heterogeneity and sensitivity to influential effects indicate that these findings should be interpreted cautiously.

**2.3.2 Moderator Analysis**

For vertical jump height, subgroup-specific pooled effects estimated from the no-intercept models suggested significant effects across multiple moderator levels in both the between-group (ISO vs. CON) and within-group (pre- vs. post-ISO) analyses, including sex, strength training experience, intensity, contraction duration, volume, rest interval, repetition configuration, intervention modality, and joint angle (Figure 8 and 9). Regarding the with-intercept models, no moderator showed a significant between-level effect in the within-group comparison. In the between-group comparison, joint angle remained a significant moderator (F = 4.325, p = 0.043), with greater effects observed for knee angles between 90° and 140° than for angles ≤ 90° (difference = 0.500, 95% CI [0.015, 0.984]). Volume also showed a significant effect (F = 4.499, p = 0.017), although individual contrasts did not reach statistical significance. All other moderators were not statistically significant in the with-intercept models (Details in Supplementary Material 6) **(Figure 8 and 9)**.

**2.3.4 Meta-regression**

For the relationship between rest interval and vertical jump within isometric contraction group, the natural cubic spline showed the best in-sample fit among the non-linear specifications (R² = 0.249; adjusted R² = 0.225; overall p = 0.00011) **(Figure 10)**. However, it provided only limited improvement over the linear model: only one spline basis term was significant (β₁ = 0.333, p < 0.001), whereas the additional curvature term was not (β₂ = 0.111, p = 0.376), and information-criterion gains were small (ΔAIC = 0.59; ΔAICc = 0.79; ΔBIC = 2.78). Given prior expectations of nonlinearity and these modest gains, we use the spline to illustrate a potential non-linear pattern—with the largest model-predicted effect at ~7.7 min (ES = 0.23)—but emphasize that evidence for nonlinearity is limited. Estimates are less stable where data are sparse (<3 and >10 min). For the relationship between contraction duration and vertical jump, the natural cubic spline showed the best in-sample fit among the linear and non-linear specifications (R² = 0.393; adjusted R² = 0.374; overall p <0.01, β₁ = 0.381, p <0.01; β₂ = −0.262, p = 0.0012) (**Figure 11**). Information-criterion comparisons favored the same model. The inverted U-shaped curve revealed the optimal improvement with contraction duration of 8.2 seconds·session^-1^ (ES=0.30). For the relationship between volume and vertical jump, the natural cubic spline showed the best in-sample fit among the linear and non-linear specifications (R² = 0.387; adjusted R² = 0.368; overall p <0.01; β₁ = 0.377, p <0.01; β₂ = −0.197, p = 0.0045) (**Figure 12).** Information-criterion comparisons favored the same model. The inverted U-shaped curve revealed the optimal improvement with volume of 7.4 unit·session^-1^ (ES=0.28). Due to the unbalanced distribution of data points in studies reporting strength and power output outcomes, meta-regression analysis was not feasible. Detailed information regarding the meta-regression results can be found in supplementary material 7.

**Reference**

1.	Garcia T, Pupo J, Knihs D, Rodrigues OF, Zimmermann H, Padulo JJHM. Single-versus multi-joint isometric protocols to induce a post activation potentiation effect on squat jump performance. 2020;21(3):71-80.

2.	Herring CH, Goldstein ER, Fukuda DHJTJoS, Research C. Use of tensiomyography in evaluating sex-based differences in resistance-trained individuals after plyometric and isometric midthigh pull postactivation potentiation protocols. 2021;35(6):1527-34.

3.	Koźlenia D. The role of stretch-shortening cycle and sex in subsequent jump tasks improvement after conditioning activity based on holding isometric muscle action. The Journal of sports medicine and physical fitness. 2024.

4.	Koźlenia D, Domaradzki J. Postsubmaximal Isometric Full Squat Jump Potentiation in Trained Men. Journal of strength and conditioning research. 2024;38(3):459-64.

5.	Koźlenia D, Domaradzki JJSR. The effectiveness of isometric protocols using an external load or voluntary effort on jump height enhancement in trained females. 2023;13(1):13535.

6.	Koźlenia D, Domaradzki JJP. Effects of post-activation protocols based on slow tempo bodyweight squat and isometric activity on vertical jump height enhancement in trained males: a randomized controlled trial. 2023;11:e15753.

7.	Krzysztofik M, Spieszny M, Trybulski R, Wilk M, Pisz A, Kolinger D, et al. Acute effects of isometric conditioning activity on the viscoelastic properties of muscles and sprint and jumping performance in handball players. 2023;37(7):1486-94.

8.	Vargas-Molina S, Salgado-Ramírez U, Chulvi-Medrano I, Carbone L, Maroto-Izquierdo S, Benítez-Porres JJPo. Comparison of post-activation performance enhancement (PAPE) after isometric and isotonic exercise on vertical jump performance. 2021;16(12):e0260866.

9.	Koźlenia D, Domaradzki JJFiP. The sex effects on changes in jump performance following an isometric back squat conditioning activity in trained adults. 2023;14:1156636.

10.	Hernández-Trujillo JA, González-Rivera MD, Romero-Franco N, González-Hernández JMJP. Acute effects of voluntary isometric contractions at maximal shortening vs. ballistic stretching on flexibility, strength and jump. 2024;12:e17819.

11.	Lima LCR, Oliveira FBD, Oliveira TP, Assumpcao CdO, Greco CC, Cardozo AC, et al. Postactivation potentiation biases maximal isometric strength assessment. 2014;2014(1):126961.

12.	Tsoukos A, Bogdanis GC, Terzis G, Veligekas P. Acute Improvement of Vertical Jump Performance After Isometric Squats Depends on Knee Angle and Vertical Jumping Ability. Journal of strength and conditioning research. 2016;30(8):2250-7.

13.	Batista MA, Roschel H, Barroso R, Ugrinowitsch C, Tricoli VJTJoS, Research C. Influence of strength training background on postactivation potentiation response. 2011;25(9):2496-502.

14.	Esformes JI, Keenan M, Moody J, Bampouras TM. Effect of different types of conditioning contraction on upper body postactivation potentiation. Journal of strength and conditioning research. 2011;25(1):143-8.

15.	French DN, Kraemer WJ, Cooke CBJTJoS, Research C. Changes in dynamic exercise performance following a sequence of preconditioning isometric muscle actions. 2003;17(4):678-85.

16.	Montano J, Torossian E, Martinez VG, Lhanie L, Awakimian S, Lopes CR, et al. Different barbell height positions affect maximal isometric deadlift force and subsequent squat jump performance in recreationally-trained men. 2021;14(4):1247.

17.	D PIPER A, Joubert DP, Jones EJ, T WHITEHEAD MJIJoES. Comparison of post-activation potentiating stimuli on jump and sprint performance. 2020;13(4):539.

18.	Robbins DW, Docherty D. Effect of loading on enhancement of power performance over three consecutive trials. Journal of strength and conditioning research. 2005;19(4):898-902.

19.	Skurvydas A, Jurgelaitiene G, Kamandulis S, Mickeviciene D, Brazaitis M, Valanciene D, et al. What are the best isometric exercises of muscle potentiation? 2019;119:1029-39.

20.	Spieszny M, Trybulski R, Biel P, Zając A, Krzysztofik M. Post-Isometric Back Squat Performance Enhancement of Squat and Countermovement Jump. International journal of environmental research and public health. 2022;19(19).

21.	Tsolakis C, Bogdanis GC, Nikolaou A, Zacharogiannis E. Influence of type of muscle contraction and gender on postactivation potentiation of upper and lower limb explosive performance in elite fencers. Journal of sports science & medicine. 2011;10(3):577-83.

22.	Downey RJ, Deprez DA, Chilibeck PDJTJoS, Research C. Effects of postactivation potentiation on maximal vertical jump performance after a conditioning contraction in upper-body and lower-body muscle groups. 2022;36(1):259-61.

23.	Ino T, Ogawara K, Uchiyama S, Endo S, Miyazaki S, Yamada HJm. Effect of maximum voluntary isometric contraction of the triceps surae muscle on a subsequent drop jump. 2024:2024.09. 04.24312972.

24.	Jarosz J, Gaweł D, Grycmann P, Ashenbrenner P, Spieszny M, Wilk M, et al. How Repeatable is PAPE Effect: The Impact of In-Season Isometric Squat Activation on Countermovement Jump Performance Enhancement in Professional Soccer Players. 2024.

25.	Rixon KP, Lamont HS, Bemben MGJTJoS, Research C. Influence of type of muscle contraction, gender, and lifting experience on postactivation potentiation performance. 2007;21(2):500-5.
