# Supplementary material for: From Populations to Programming Variables: Post‐Activation Performance Enhancement Following Isometric Contractions on Muscular Strength and Power—A Systematic Review and Meta‐Analysis
Source: Eur J Sport Sci. 2026 May 19;26(6):e70183. doi: 10.1002/ejsc.70183 (PMC13580768; doi:10.1002/ejsc.70183)
Supplement: Supplementary file 3 [file EJSC-26-e70183-s003.docx]

**The search string is as follows:**

(((((((isometric*[Title/Abstract]) OR (static[Title/Abstract])) OR (maximal voluntary contraction[Title/Abstract])) OR (MVC[Title/Abstract])) OR (PIMA[Title/Abstract])) OR (HIMA[Title/Abstract])) OR (IMTP[Title/Abstract])) OR (isometric mid-thigh pull[Title/Abstract]) AND (((((((((post-activation potentiation[Title/Abstract]) OR (PAP[Title/Abstract])) OR (post-activation performance enhancement[Title/Abstract])) OR (PAPE[Title/Abstract])) OR (conditioning activit*[Title/Abstract])) OR (CA[Title/Abstract])) OR (warm-up*[Title/Abstract])) OR (priming[Title/Abstract])) OR (acute enhancement[Title/Abstract])) OR (acute improvement[Title/Abstract])
