# Supplementary material for: From Populations to Programming Variables: Post‐Activation Performance Enhancement Following Isometric Contractions on Muscular Strength and Power—A Systematic Review and Meta‐Analysis
Source: Eur J Sport Sci. 2026 May 19;26(6):e70183. doi: 10.1002/ejsc.70183 (PMC13580768; doi:10.1002/ejsc.70183)
Supplement: Supplementary file 4 [file EJSC-26-e70183-s004.docx]

**Study quality of the included studies was evaluated using the modified PEDro scale, as adapted by Brughelli et al:**

The evaluation focused on the following criteria: (1) clarity of inclusion criteria, (2) random allocation of participants to groups, (3) clear definition of interventions, (4) baseline group similarity, (5) inclusion of a control condition, (6) clear definition of outcome variables, (7) practical utility of assessments, (8) practical duration of intervention, (9) appropriateness of between-group statistical analyses, and (10) provision of point measures of variability. The scale consists of 10 items, with a total score range of 0 to 20. Scores for each criterion were assigned as follows: 0 = clearly no, 1 = maybe, and 2 = clearly yes.
