# Supplementary material for: From Populations to Programming Variables: Post‐Activation Performance Enhancement Following Isometric Contractions on Muscular Strength and Power—A Systematic Review and Meta‐Analysis
Source: Eur J Sport Sci. 2026 May 19;26(6):e70183. doi: 10.1002/ejsc.70183 (PMC13580768; doi:10.1002/ejsc.70183)
Supplement: Supplementary file 5 [file EJSC-26-e70183-s005.docx]

**Assessment of Risk of Bias and Study Quality**

A modified version of the Cochrane Collaboration tool was utilized to assess the methodological quality of the included studies(1). Two aspects (Allocation concealment and blinding of implementers) were excluded due to the challenges of implementation in PAPE studies (2). Four PAPE-specific domains were included to better capture sources of bias that are particularly relevant to acute conditioning activity studies, namely cumulative effects from repeated post-CA testing, measurement bias, sample size bias, and test familiarization. (2). Specifically, cumulative effect bias was assessed according to the number and spacing of post-CA tests performed. This operational criterion was adapted from a recent PAPE-specific meta-analysis **(2)**, in which cumulative-effect bias was incorporated as an additional domain within a modified Cochrane tool and justified with reference to phosphocreatine recovery time **(3, 4)**. Studies were classified as “low risk” when only one post-CA test (i.e., one time point) was conducted, as “unclear risk” when two or more post-CA tests were performed over a time interval of at least 4 min or when multiple tests were conducted simultaneously, and as “high risk” when two or more post-CA tests were conducted with an inter-test interval of < 4 min. We acknowledge that this classification should be regarded as a pragmatic PAPE-specific operational rule rather than a universally validated risk-of-bias standard. Measurement bias was evaluated based on the reporting of reliability statistics. Studies were considered “low risk” when two or more reliability indices were reported (e.g., intraclass correlation coefficient [ICC] and coefficient of variation [CV]), “unclear risk” when only one reliability statistic was reported, and “high risk” when no reliability statistic was provided. Sample size bias was assessed according to whether an a priori sample size estimation was performed and correctly described. Studies were rated as “low risk” when an a priori sample size estimation was appropriately reported, including correct distinction between effect size estimates for t-tests and F-tests. Studies were rated as “unclear risk” when no such procedure was reported. Studies were rated as “high risk” when no a priori sample size estimation was reported and the sample size was < 10 participants, or when effect sizes were estimated incorrectly. Finally, test familiarization was evaluated based on whether familiarization sessions were conducted and, if so, how many. Studies were classified as “low risk” when ≥ 2 familiarization sessions were completed, as “unclear risk” when only one familiarization session was conducted, and as “high risk” when no familiarization session was reported.

1.	Higgins JJTCC. The Cochrane Collaboration’s Tool for Assessing Risk of Bias in Randomised Trials. 2011.

2.	Xu K, Blazevich A, Boullosa D, Ramirez-Campillo R, Yin M, Zhong Y, et al. Optimizing post-activation performance enhancement in athletic tasks: A systematic review with meta-analysis for prescription variables and research methods. Sports Medicine. 2024.

3.	McMahon S, Jenkins D. Factors affecting the rate of phosphocreatine resynthesis following intense exercise. Sports medicine (Auckland, NZ). 2002;32(12):761–84.

4.	Jones AM, Wilkerson DP, Berger NJ, Fulford J. Influence of endurance training on muscle [PCr] kinetics during high-intensity exercise. American journal of physiology Regulatory, integrative and comparative physiology. 2007;293(1):R392–401.
