# Supplementary material for: From Populations to Programming Variables: Post‐Activation Performance Enhancement Following Isometric Contractions on Muscular Strength and Power—A Systematic Review and Meta‐Analysis
Source: Eur J Sport Sci. 2026 May 19;26(6):e70183. doi: 10.1002/ejsc.70183 (PMC13580768; doi:10.1002/ejsc.70183)
Supplement: Supplementary file 6 [file EJSC-26-e70183-s006.docx]

**Participant Characteristics**

The effects of PAPE are influenced by participants’ strength training experience and strength levels [1]. Most included studies involved strength trained individuals. To meet statistical requirements for moderator analysis and based on the distribution of available data, we categorized training experience into two groups for vertical jump and power outcomes: “<2 years” (including untrained individuals and those with less than two years of experience) and “≥2 years”. For strength outcomes, participants were classified as either “strength trained” or “strength untrained”. This distinction was necessary because 47.4% of the included studies did not report detailed strength levels, and most reported only training experience ranges or thresholds. For studies lacking precise data, the minimum reported training duration was used for categorization (e.g., “at least one year” was categorized as “<2 years”). This approach is consistent with current PAPE literature recommendations [1, 2].

**Intervention Variables**

Contraction duration was calculated as the total number of seconds per intervention session (sustained contraction duration per repetition × repetitions × sets (42)); Published studies on acute isometric interventions lack consensus on classification standards for contraction duration(49, 50). Volume was defined as the intensity-weighted contraction stimulus within one intervention session, based on traditional dynamic strength training concepts (51). Because no clear operational definition of training volume currently exists in the isometric training literature (19, 40) , session volume was calculated using the following equation:

*volume = relative intensity × contraction duration*
where maximal intensity was coded as 1.0 and submaximal intensity was expressed as a decimal (e.g., 80% MVC or 80% 1RM = 0.80). Accordingly, one volume unit represented 1 s of maximal isometric contraction, or the equivalent intensity-weighted contraction stimulus under submaximal conditions. For example, 3 repetitions of 5-s isometric contractions performed at 80% MVC corresponded to 12 volume units. Intervention modality was categorized as HIMA (Holding Isometric Muscle Action, maintaining a set position while resisting an external force) and PIMA (Pushing Isometric Muscle Action, involving pushing or pulling against an immovable object) [7]; Repetition configuration was grouped as “single rep” or “multiple reps” to control for design heterogeneity (some studies prescribed one set with only one rep, while others prescribed one set with multiple reps, potentially leading to distinct effects when categorized simply as one set versus multiple sets). For joint angle, studies showed that isometric contraction at different joint angles (e.g., 80° vs. 130°) may produce distinct stimuli and neuromuscular adaptations [9-11]. Considering that most included studies focused on lower-limb isometrics and reported knee angles, 68.12% of the included studies reported knee angles ≤90°, 14.49% reported 91-140°, and 17.39% were unclear. We categorized knee angles as “knee angle ≤90°”, “90° < knee angle ≤140°”, and “unclear”. For studies that did not report the exact knee angle, categorization was based on the study description (e.g., studies reporting isometric squats with the femur parallel to the ground were categorized as “knee angle ≤90°”). No included studies were difficult to classify into these groups. Rest interval groups were selected as following because studies on PAPE suggested that a rest period of at least 4 minutes between squats and subsequent performance is effective [1, 12, 13], as the phosphagen system is nearly fully recovered at 4 minutes [14], and the meta-analysis by Seitz et al. found different effects of PAPE between rest intervals with thresholds of 4 and 8 minutes [1].

1.	Seitz LB, Haff GG. Factors Modulating Post-Activation Potentiation of Jump, Sprint, Throw, and Upper-Body Ballistic Performances: A Systematic Review with Meta-Analysis. Sports medicine (Auckland, NZ). 2016;46(2):231-40.

2.	Xu K, Blazevich A, Boullosa D, Ramirez-Campillo R, Yin M, Zhong Y, et al. Optimizing post-activation performance enhancement in athletic tasks: A systematic review with meta-analysis for prescription variables and research methods. Sports Medicine. 2024.

3.	Lum D, Barbosa TM. Brief Review: Effects of Isometric Strength Training on Strength and Dynamic Performance. International journal of sports medicine. 2019;40(6):363-75.

4.	Kubo K, Kanehisa H, Fukunaga T. Effects of different duration isometric contractions on tendon elasticity in human quadriceps muscles. The Journal of physiology. 2001;536(Pt 2):649-55.

5.	Kanehisa H, Nagareda H, Kawakami Y, Akima H, Masani K, Kouzaki M, et al. Effects of equivolume isometric training programs comprising medium or high resistance on muscle size and strength. European journal of applied physiology. 2002;87(2):112-9.

6.	Carvalho L, Junior RM, Barreira J, Schoenfeld BJ, Orazem J, Barroso R. Muscle hypertrophy and strength gains after resistance training with different volume-matched loads: a systematic review and meta-analysis. Applied physiology, nutrition, and metabolism = Physiologie appliquee, nutrition et metabolisme. 2022;47(4):357-68.

7.	Oranchuk DJ, Nelson AR, Lum D, Natera AO, Bittmann FN, Schaefer LVJm. ‘Pushing’versus ‘holding’isometric muscle actions; what we know and where to go: A scoping and systematic review with meta-analyses. 2024:2024.11. 04.24316609.

8.	Oranchuk DJ, Storey AG, Nelson AR, Cronin JB. Isometric training and long-term adaptations: Effects of muscle length, intensity, and intent: A systematic review. Scandinavian journal of medicine & science in sports. 2019;29(4):484-503.

9.	Kubo K, Ohgo K, Takeishi R, Yoshinaga K, Tsunoda N, Kanehisa H, et al. Effects of isometric training at different knee angles on the muscle-tendon complex in vivo. Scandinavian journal of medicine & science in sports. 2006;16(3):159-67.

10.	Noorkõiv M, Nosaka K, Blazevich AJ. Effects of isometric quadriceps strength training at different muscle lengths on dynamic torque production. Journal of sports sciences. 2015;33(18):1952-61.

11.	Alegre LM, Ferri-Morales A, Rodriguez-Casares R, Aguado X. Effects of isometric training on the knee extensor moment-angle relationship and vastus lateralis muscle architecture. European journal of applied physiology. 2014;114(11):2437-46.

12.	Jensen RL, Ebben WP. Kinetic analysis of complex training rest interval effect on vertical jump performance. Journal of strength and conditioning research. 2003;17(2):345-9.

13.	Comyns TM, Harrison AJ, Hennessy LK, Jensen RL. The optimal complex training rest interval for athletes from anaerobic sports. Journal of strength and conditioning research. 2006;20(3):471-6.

14.	Ellington WR. Evolution and physiological roles of phosphagen systems. Annual review of physiology. 2001;63:289-325.
