# Supplementary material for: From Populations to Programming Variables: Post‐Activation Performance Enhancement Following Isometric Contractions on Muscular Strength and Power—A Systematic Review and Meta‐Analysis
Source: Eur J Sport Sci. 2026 May 19;26(6):e70183. doi: 10.1002/ejsc.70183 (PMC13580768; doi:10.1002/ejsc.70183)
Supplement: Supplementary file 7 [file EJSC-26-e70183-s007.docx]

| **Table 6.1 GRADE Assessment for the Certainty of Evidence for Vertical Jump (ISO-Pre vs Post)** | | | | | | | | | |
| --- | --- | --- | --- | --- | --- | --- | --- | --- | --- |
| Certainty assessment | | | | | | | | | |
| Moderators | No. of studies | No. of effect sizes | Risk of bias | Inconsistency | Indirectness | Imprecision | Publication bias | Effect Size [95%CI] | Certainty |
| Volume | | | | | | | | | |
| Low | 5 | 11 | Serious | Not serious | Not serious | Serious | Not serious | 0.16  [－0.04, 0.37] | ⊕⊕○○  Low |
| Moderate | 8 | 39 | Not serious | Not serious | Not serious | Serious | Not serious | 0.26  [0.14, 0.37] | ⊕⊕⊕○  Moderate |
| High | 5 | 16 | Serious | Serious | Not serious | Serious | Not serious | －0.02  [－0.23, 0.18] | ⊕⊕○○  Low |
| Intensity | | | | | | | |  |  |
| Submaximal | 6 | 23 | Not serious | Serious | Not serious | Serious | Not serious | 0.26  [0.09, 0.43] | ⊕⊕○○  Low |
| Maximal | 10 | 43 | Serious | Serious | Not serious | Serious | Not serious | 0.13  [0, 0.27] | ⊕⊕○○  Low |
| Duration | | | | | | | |  |  |
| Long | 8 | 36 | Serious | Not Serious | Not serious | Serious | Not serious | 0.16  [0, 0.32] | ⊕⊕○○  Low |
| Moderate | 5 | 20 | Serious | Not Serious | Not serious | Serious | Not serious | 0.22  [0.02, 0.43] | ⊕⊕○○  Low |
| Short | 5 | 11 | Serious | Not Serious | Not serious | Serious | Not serious | 0.16  [－0.08, 0.40] | ⊕⊕○○  Low |
| Interval | | | | | | | | | |
| Long | 7 | 12 | Not serious | Serious | Not serious | Serious | Not serious | 0.21  [－0.02, 0.43] | ⊕⊕○○  Low |
| Moderate | 11 | 31 | Serious | Not serious | Not serious | Serious | Not serious | 0.18  [0.04, 0.32] | ⊕⊕○○  Low |
| Short | 10 | 24 | Serious | Serious | Not serious | Serious | Not serious | 0.17  [0.02, 0.32] | ⊕⊕○○  Low |
| Strength Training Experience | | | | | | | | | |
| ≥2years | 12 | 59 | Serious | Not serious | Not serious | Serious | Not serious | 0.19  [0.08, 0.30] | ⊕⊕○○  Low |
| ＜2years | 5 | 8 | Serious | Not serious | Not serious | Serious | Not serious | 0.10  [－0.09, 0.44] | ⊕⊕○○  Low |
| Reps | | | | | | | | | |
| Multi | 13 | 57 | Serious | Not serious | Not serious | Serious | Not serious | 0.19  [0.07, 0.30] | ⊕⊕○○  Low |
| Single | 4 | 10 | Serious | Not serious | Not serious | Serious | Not serious | 0.15  [－0.09, 0.39] | ⊕⊕○○  Low |
| Intervention Modality | | | | | | | | | |
| HIMA | 6 | 23 | Serious | Serious | Not serious | Serious | Not serious | 0.26  [0.09, 0.42] | ⊕⊕○○  Low |
| PIMA | 11 | 44 | Serious | Not serious | Not serious | Serious | Not serious | 0.14  [0.01, 0.26] | ⊕⊕○○  Low |
| Gender | | | | | | | | | |
| Female | 3 | 14 | Serious | Serious | Not serious | Serious | Not serious | 0.03  [－0.19, 0.26] | ⊕⊕○○  Low |
| Male | 12 | 49 | Serious | Not serious | Not serious | Serious | Not serious | 0.22  [0.11, 0.33] | ⊕⊕○○  Low |
| Joint Angle | | | | | | | | | |
| 90°-140° | 4 | 10 | Serious | Serious | Not serious | Serious | Not serious | 0.33  [0.07, 0.60] | ⊕⊕○○  Low |
| ≤90° | 11 | 52 | Serious | Not serious | Not serious | Serious | Not serious | 0.13  [0, 0.27] | ⊕⊕○○  Low |
| Overall | 16 | 67 | Serious | Not serious | Not serious | Serious | Not serious | 0.18  [0.08, 0.28] | ⊕⊕○○  Low |

| **Table 6.2 GRADE Assessment for the Certainty of Evidence for Vertical Jump Iso vs Con** | | | | | | | | | |
| --- | --- | --- | --- | --- | --- | --- | --- | --- | --- |
| Certainty assessment | | | | | | | | | |
| Moderators | No. of studies | No. of effect sizes | Risk of bias | Inconsistency | Indirectness | Imprecision | Publication bias | Effect Size [95%CI] | Certainty |
| Volume | | | | | | | | | |
| Low | 2 | 5 | Serious | Not serious | Not serious | Serious | Not serious | 0.09  [－0.40, 0.58] | ⊕⊕○○  Low |
| Moderate | 7 | 31 | Not serious | Serious | Not serious | Serious | Not serious | 0.55  [0.34, 0.76] | ⊕⊕○○  Low |
| High | 5 | 14 | Serious | Serious | Not serious | Serious | Not serious | 0.08  [－0.22, 0.37] | ⊕⊕○○  Low |
| Intensity | | | | | | | | | |
| Submaximal | 4 | 15 | Serious | Serious | Not serious | Serious | Not serious | 0.59  [0.25, 0.92] | ⊕⊕○○  Low |
| Maximal | 8 | 35 | Serious | Serious | Not serious | Serious | Not serious | 0.25  [0, 0.49] | ⊕⊕○○  Low |
| Duration | | | | | | | | | |
| Long | 8 | 27 | Serious | Serious | Not serious | Serious | Not serious | 0.34  [0.03, 0.66] | ⊕⊕○○  Low |
| Moderate | 4 | 18 | Not serious | Not serious | Not serious | Serious | Not serious | 0.42  [0.01, 0.82] | ⊕⊕⊕○  Moderate |
| Short | 2 | 5 | Serious | Not serious | Not serious | Serious | Not serious | 0.20  [－0.39, 0.79] | ⊕⊕○○  Low |
| Interval | | | | | | | | | |
| Long | 6 | 10 | Serious | Serious | Not serious | Serious | Not serious | 0.46  [0.10, 0.81] | ⊕⊕○○  Low |
| Moderate | 10 | 30 | Serious | Serious | Not serious | Serious | Not serious | 0.40  [0.13, 0.66] | ⊕⊕○○  Low |
| Short | 5 | 11 | Serious | Serious | Not serious | Serious | Not serious | 0.10  [－0.24, 0.44] | ⊕⊕○○  Low |
| Strength Training Experience | | | | | | | | | |
| ≥2 years | 10 | 46 | Serious | Serious | Not serious | Serious | Not serious | 0.37  [0.13, 0.62] | ⊕⊕○○  Low |
| ＜2 years | 3 | 5 | Serious | Not serious | Not serious | Serious | Not serious | 0.18  [－0.45, 0.80] | ⊕⊕○○  Low |
| Reps | | | | | | | | | |
| Multi | 11 | 46 | Serious | Serious | Not serious | Serious | Not serious | 0.37  [0.14, 0.60] | ⊕⊕○○  Low |
| Single | 2 | 5 | Serious | Not serious | Not serious | Serious | Not serious | 0.19  [－0.37, 0.75] | ⊕⊕○○  Low |
| Intervention Modality | | | | | | | | | |
| HIMA | 4 | 15 | Serious | Serious | Not serious | Serious | Not serious | 0.59  [0.26, 0.91] | ⊕⊕○○  Low |
| PIMA | 9 | 36 | Serious | Serious | Not serious | Serious | Not serious | 0.25  [0.02, 0.48] | ⊕⊕○○  Low |
| Gender | | | | | | | | | |
| Female | 3 | 11 | Not serious | Serious | Not serious | Serious | Not serious | 0.38  [－0.14, 0.89] | ⊕⊕○○  Low |
| Male | 9 | 37 | Serious | Serious | Not serious | Serious | Not serious | 0.36  [0.08, 0.65] | ⊕⊕○○  Low |
| Joint Angle | | | | | | | | | |
| 90°-140° | 2 | 7 | Serious | Serious | Not serious | Serious | Not serious | 0.80  [0.30, 1.31] | ⊕⊕○○  Low |
| ≤90° | 9 | 41 | Serious | Serious | Not serious | Serious | Not serious | 0.30  [0.02, 0.59] | ⊕⊕○○  Low |
| Overall | 12 | 51 | Serious | Not serious | Not serious | Serious | Not serious | 0.35  [0.13, 0.57] | ⊕⊕○○  Low |

| **Table 6.3 GRADE Assessment for the Certainty of Evidence for Vertical Jump (ISO vs DYN)** | | | | | | | | |
| --- | --- | --- | --- | --- | --- | --- | --- | --- |
| No. of studies | No. of Effect sizes | Risk of bias | Inconsistency | Indirectness | Imprecision | Publication bias | Effect Size （95%CI） | Certainty |
| 3 | 9 | serious | not serious | not serious | serious | not serious | ES －0.06 （－0.35 to 0.23） | ⊕⊕○○ Low |
|  |  |  |  |  |  |  |  |  |

| **Table 6.4 GRADE Assessment for the Certainty of Evidence for Muscular Strength (ISO-Pre vs Post)** | | | | | | | | | |
| --- | --- | --- | --- | --- | --- | --- | --- | --- | --- |
| Certainty assessment | | | | | | | | | |
| Moderators | No. of studies | No. of effect sizes | Risk of bias | Inconsistency | Indirectness | Imprecision | Publication bias | Effect Size [95%CI] | Certainty |
| Overall | 7 | 58 | Serious | Serious | Serious | Serious | Not serious | 0.23  [－0.39, 0.85] | ⊕⊕○○  Low |

| **Table 6.5 GRADE Assessment for the Certainty of Evidence for Muscular Strength (ISO vs CON)** | | | | | | | | |
| --- | --- | --- | --- | --- | --- | --- | --- | --- |
| No. of studies | No. of Effect sizes | Risk of bias | Inconsistency | Indirectness | Imprecision | Publication bias | Effect Size （95%CI） | Certainty |
| 4 | 10 | serious | serious | not serious | serious | not serious | 0.54  [－0.22, 1.30] | ⊕⊕○○  Low |
|  |  |  |  |  |  |  |  |  |

| **Table 6.6 GRADE Assessment for the Certainty of Evidence for Muscular Power (ISO-Pre vs Post)** | | | | | | | | | |
| --- | --- | --- | --- | --- | --- | --- | --- | --- | --- |
| Certainty assessment | | | | | | | | | |
| Moderators | No. of studies | No. of effect sizes | Risk of bias | Inconsistency | Indirectness | Imprecision | Publication bias | Effect Size [95%CI] | Certainty |
| Overall | 4 | 27 | Serious | Serious | Serious | Serious | Not serious | －0.13  [－0.78, 0.52] | ⊕⊕○○  Low |
