# Supplementary material for: From Populations to Programming Variables: Post‐Activation Performance Enhancement Following Isometric Contractions on Muscular Strength and Power—A Systematic Review and Meta‐Analysis
Source: Eur J Sport Sci. 2026 May 19;26(6):e70183. doi: 10.1002/ejsc.70183 (PMC13580768; doi:10.1002/ejsc.70183)
Supplement: Supplementary file 8 [file EJSC-26-e70183-s008.docx]

If the original data were not reported or were incomplete, the corresponding authors of the included studies were contacted directly. If no response was received (up to January 5, 2025) or if the authors declined to provide the original data, the following actions were taken:

(1) For studies that did not report outcomes as "Mean±SD (standard deviation)" or where outcomes were presented as "Mean±SE/SEM (standard error/standard error of mean)", calculations were performed using the following formulas:

$$\boldsymbol{Mean}_{\boldsymbol{diff}}\boldsymbol{=}\boldsymbol{Mean}_{\boldsymbol{2}}\boldsymbol{-}\boldsymbol{Mean}_{\boldsymbol{1}}\boldsymbol{;}\boldsymbol{SD}_{\boldsymbol{diff}}\boldsymbol{=}\sqrt{\boldsymbol{SD}_{\boldsymbol{1}}^{\boldsymbol{2}}\boldsymbol{+}\boldsymbol{SD}_{\boldsymbol{2}}^{\boldsymbol{2}}\boldsymbol{-}\boldsymbol{2}\boldsymbol{\times SD}_{\boldsymbol{1}}\boldsymbol{\times}\boldsymbol{SD}_{\boldsymbol{2}}\boldsymbol{\times R}}$$

$$\boldsymbol{SD}_{\boldsymbol{1}}\boldsymbol{=}\boldsymbol{SE}_{\boldsymbol{1}}\boldsymbol{\times}\sqrt{\boldsymbol{n}}\boldsymbol{;SD}_{\boldsymbol{2}}\boldsymbol{=}\boldsymbol{SE}_{\boldsymbol{2}}\boldsymbol{\times}\sqrt{\boldsymbol{n}}$$

(2) For studies that only reported the mean change and standard deviation (SD) of the change within groups, as well as either pre-test or post-test data, the missing pre-test or post-test means and SDs were calculated using the following methods:

Calculating Post-test Mean and SD from Pre-test Data:

$$\boldsymbol{M}_{\boldsymbol{post}}\boldsymbol{=}\boldsymbol{M}_{\boldsymbol{pre}}\boldsymbol{+ \Delta M}$$

$$\boldsymbol{SD}_{\boldsymbol{post}}\boldsymbol{=}\sqrt{\boldsymbol{SD}_{\boldsymbol{pre}}^{\boldsymbol{2}}\boldsymbol{+}\boldsymbol{SD}_{\boldsymbol{\Delta M}}^{\boldsymbol{2}}\boldsymbol{-}\boldsymbol{2}{\times\boldsymbol{SD}}_{\boldsymbol{pre}}\boldsymbol{\times}\boldsymbol{SD}_{\boldsymbol{\Delta M}}\boldsymbol{\times R}}$$

Calculating Pre-test Mean and SD from Post-test Data:

$$\boldsymbol{M}_{\boldsymbol{pre}}\boldsymbol{=}\boldsymbol{M}_{\boldsymbol{post}}\boldsymbol{- \Delta M}$$

$$\boldsymbol{SD}_{\boldsymbol{pre}}\boldsymbol{=}\sqrt{\boldsymbol{SD}_{\boldsymbol{post}}^{\boldsymbol{2}}\boldsymbol{+}\boldsymbol{SD}_{\boldsymbol{\Delta M}}^{\boldsymbol{2}}\boldsymbol{-}\boldsymbol{2}{\times\boldsymbol{SD}}_{\boldsymbol{post}}\boldsymbol{\times}\boldsymbol{SD}_{\boldsymbol{\Delta M}}\boldsymbol{\times R}}$$

Note: R = 0.5; SD_1_, SD_2_ = standard deviation of pre and post; SE_1_, SE_2_ = standard error of pre and post; Mean2, Mean1 = Mean of pre and post.
