# Supplementary material for: From Populations to Programming Variables: Post‐Activation Performance Enhancement Following Isometric Contractions on Muscular Strength and Power—A Systematic Review and Meta‐Analysis
Source: Eur J Sport Sci. 2026 May 19;26(6):e70183. doi: 10.1002/ejsc.70183 (PMC13580768; doi:10.1002/ejsc.70183)
Supplement: Supplementary file 9 [file EJSC-26-e70183-s009.docx]

| **Table** Summary of moderator analysis on vertical jump height (no intercepts) | | | | | | | | | | | | | | | |
| --- | --- | --- | --- | --- | --- | --- | --- | --- | --- | --- | --- | --- | --- | --- | --- |
| **PRE VS POST within Isometric Group on Vertical Jump Height (no intercepts)** | | | | | | | | | | | | | | | |
| **Moderator** | **Moderator-Level** | **Original Model** | | | | | | | **Model without Influential Studies** | | | | | | |
|  |  | ***I^2^*** | | **ES [95%CI]** | ***p*-value** | ***t*-value** | **Test of Moderator ^a^** | | ***I^2^*** | | **ES [95%CI]** | ***p*-value** | ***t*-value** | **Test of Moderator ^a^** | |
|  |  | ***l_2_*** | ***l_3_*** |  |  |  | ***F*** | ***p*** | ***l_2_*** | ***L_3_*** |  |  |  | ***F*** | ***p*** |
| **Volume^*^** | Low  (k=5, n=11) | **0%** | **4.47%** | **0.205 [-0.001, 0.411]** | **0.051** | **1.988** | **6.556** | **0.0006** | **0%** | **2.48%** | **0.161 [-0.044, 0.367]** | **0.122** | **1.568** | **7.419** | **0.0003** |
|  | Moderate^#^  (k=8, n=38) |  |  | **0.236 [0.115, 0.358]** | **0.0003** | **3.871** |  |  |  |  | **0.257 [0.141, 0.372]** | **<0.0001** | **4.441** |  |  |
|  | High  (k=5, n=16) |  |  | **-0.075 [-0.272, 0.122]** | **0.451** | **-0.758** |  |  |  |  | **-0.0230 [-0.229, 0.184]** | **0.825** | **-0.222** |  |  |
| **Intensity^*^** | Submaximal Intensity^#^  (k=6, n=23) | **0%** | **10.95%** | **0.254 [0.0767, 0.432]** | **0.006** | **2.857** | **5.197** | **0.008** | **0%** | **8.49%** | **0.256 [0.087, 0.425]** | **0.004** | **3.033** | **6.480** | **0.003** |
|  | Maximal Intensity^#^  (k=10, n=42) |  |  | **0.105 [-0.026, 0.237]** | **0.114** | **1.601** |  |  |  |  | **0.134 [0.002, 0.265]** | **0.047** | **2.031** |  |  |
| **Contraction Duration^*^** | Short Duration  (k=5, n=11) | **0%** | **17.31%** | **0.214 [-0.031, 0.459]** | **0.086** | **1.743** | **2.378** | **0.078** | **0%** | **11.46%** | **0.158 [-0.079, 0.395]** | **0.188** | **1.332** | **3.443** | **0.022** |
|  | Moderate Duration^#^  (k=5, n=19) |  |  | **0.187 [-0.037, 0.411]** | **0.100** | **1.666** |  |  |  |  | **0.224 [0.021, 0.428]** | **0.031** | **2.204** |  |  |
|  | Long Duration  (k=8, n=36) |  |  | **0.103 [-0.068, 0.275]** | **0.234** | **1.201** |  |  |  |  | **0.156 [-0.004, 0.316]** | **0.056** | **1.952** |  |  |
| **Rest Interval^*^** | Short Interval^#^  (k=10, n=24) | **0%** | **11.99%** | **0.184 [0.029, 0.340]** | **0.021** | **2.362** | **2.865** | **0.043** | **0%** | **8.58%** | **0.168 [0.016, 0.320]** | **0.031** | **2.208** | **3.967** | **0.012** |
|  | Moderate Interval^#^  (k=11, n=31) |  |  | **0.141 [-0.004, 0.286]** | **0.056** | **1.941** |  |  |  |  | **0.179 [0.036, 0.322]** | **0.015** | **2.495** |  |  |
|  | Long Interval  (k=7, n=11) |  |  | **0.135 [-0.088, 0.357]** | **0.231** | **1.209** |  |  |  |  | **0.207 [-0.016, 0.431]** | **0.069** | **1.852** |  |  |
| **Strength Training Experience^*^** | ＜2 years  (k=5, n=8) | **0%** | **12.95%** | **0.175 [-0.092, 0.443]** | **0.195** | **1.309** | **4.010** | **0.023** | **0%** | **7.66%** | **0.095 [-0.176, 0.367]** | **0.485** | **0.703** | **6.418** | **0.003** |
|  | ≥2 years^#^  (k=12, n=58) |  |  | **0.152 [0.032, 0.272]** | **0.014** | **2.527** |  |  |  |  | **0.193 [0.084, 0.303]** | **0.001** | **3.518** |  |  |
| **Rep Configuration^*^** | Multi Reps^#^  (k=13, n=56) | **0%** | **13.77%** | **0.142 [0.018, 0.266]** | **0.026** | **2.282** | **3.977** | **0.023** | **0%** | **9.03%** | **0.185 [0.069, 0.301]** | **0.002** | **3.184** | **5.803** | **0.005** |
|  | Single Rep  (k=4, n=10) |  |  | **0.210 [-0.038, 0.458]** | **0.096** | **1.688** |  |  |  |  | **0.150 [-0.092, 0.392]** | **0.220** | **1.238** |  |  |
| **Intervention Modality^*^** | PIMA^#^  (k=11, n=43) | **0%** | **10.22%** | **0.108 [-0.020, 0.235]** | **0.096** | **1.690** | **5.481** | **0.006** | **0%** | **7.92%** | **0.135 [0.008, 0.263]** | **0.038** | **2.117** | **6.790** | **0.002** |
|  | HIMA^#^  (k=6, n=23) |  |  | **0.255 [0.080, 0.429]** | **0.005** | **2.905** |  |  |  |  | **0.256 [0.090, 0.422]** | **0.003** | **3.074** |  |  |
| **Sex^*^** | Male^#^  (k=12, n=48) | **0%** | **10.3%** | **0.196 [0.077, 0.314]** | **0.002** | **3.300** | **5.5171** | **0.006** | **0%** | **5.18%** | **0.220 [0.110, 0.329]** | **0.0002** | **4.008** | **8.034** | **0.001** |
|  | Female  (k=3, n=14) |  |  | **-0.002 [-0.232, 0.229]** | **0.989** | **-0.013** |  |  |  |  | **0.034 [-0.188, 0.256]** | **0.758** | **0.310** |  |  |
| **Joint Angle^*^** | ≤90°  (k=11, n=51) | **0%** | **17.09%** | **0.107 [-0.036, 0.249]** | **0.139** | **1.501** | **4.489** | **0.015** | **0%** | **14.09%** | **0.132 [-0.003, 0.268]** | **0.056** | **1.950** | **4.683** | **0.013** |
|  | 90°＜Angle ≤140°^#^  (k=4, n=10) |  |  | **0.372 [0.100, 0.644]** | **0.008** | **2.733** |  |  |  |  | **0.332 [0.066, 0.598]** | **0.015** | **2.498** |  |  |
| **Overall** |  | **0%** | **11.65%** | **0.157 [0.049, 0.264]** | **0.005** | **2.912** |  |  | **0%** | **7.67%** | **0.180 [0.078, 0.282]** | **0.001** | **3.518** |  |  |
| **Prediction Interval** |  |  |  | **[-0.114, 0.427]** |  |  |  |  |  |  | **[-0.041, 0.401]** |  |  |  |  |

***CI*** confidence interval**; ^*^** Test of moderator reached significance in model without influential effect sizes**; ^#^** Moderator levels reached significance in model without influential effect sizes**; *l^2^*** level 2**; *l^3^*** level 3**; *ES*** effect size**; *Rep*** repetition**; *HIMA*** holding isometric muscle action**; *PIMA*** pushing isometric muscle action

| **Table** Summary of moderator analysis on vertical jump height (with intercepts) | | | | | | | | | | | | | | | |
| --- | --- | --- | --- | --- | --- | --- | --- | --- | --- | --- | --- | --- | --- | --- | --- |
| **PRE VS POST within Isometric Group on Vertical Jump height (with intercepts)** | | | | | | | | | | | | | | | |
| **Moderator** | **M****oderator-Level** | **Original Model** | | | | | | | **Model without Influential Studies** | | | | | | |
|  |  | ***I^2^*** | | **ES [95%CI]** | **p-value** | **t-value** | **Test of Moderator** | | **I****^2^** | | **ES [95%CI]** | **p-value** | **t-value** | **Test of Moderator** | |
|  |  | ***l_2_*** | ***l_3_*** |  |  |  | ***F*** | ***p*** | ***l******2*** | ***L3*** |  |  |  | ***F*** | ***p*** |
| **Contraction Duration** | Intercept  (Short, k=5, n=11) | **0** | **17.31%** | **0.214 [-0.031, 0.459]** | **0.086** | **1.743** | **0.344** | **0.711** | **0** | **11.46%** | **0.158 [-0.079, 0.395]** | **0.188** | **1.332** | **0.156** | **0.856** |
|  | Moderate  (k=5, n=19) |  |  | **-0.027 [-0.359, 0.305]** | **0.873** | **-0.161** |  |  |  |  | **0.067 [-0.246, 0.379]** | **0.672** | **0.426** |  |  |
|  | Long  (k=8, n=36) |  |  | **-0.111 [-0.407, 0.186]** | **0.459** | **-0.745** |  |  |  |  | **-0.002 [-0.286, 0.282]** | **0.990** | **-0.013** |  |  |
| **Rest Interval** | Intercept  (Short, k=10, n=24) | **0** | **11.99%** | **0.184 [0.029, 0.340]** | **0.021** | **2.362** | **0.125** | **0.883** | **0** | **8.58%** | **0.168 [0.016, 0.320]** | **0.031** | **2.208** | **0.047** | **0.954** |
|  | Moderate  (k=11, n=31) |  |  | **-0.043 [-0.239, 0.152]** | **0.659** | **-0.443** |  |  |  |  | **0.011 [-0.186, 0.207]** | **0.915** | **0.107** |  |  |
|  | Long  (k=7, n=11) |  |  | **-0.050 [-0.301, 0.202]** | **0.695** | **-0.394** |  |  |  |  | **0.039 [-0.217, 0.295]** | **0.761** | **0.305** |  |  |
| **Strength Training Experience** | Intercept  (＜2 years, k=5, n=8) | **0** | **12.95%** | **0.175 [-0.092, 0.443]** | **0.195** | **1.309** | **0.026** | **0.874** | **0** | **7.66%** | **0.095 [-0.176, 0.367]** | **0.485** | **0.703** | **0.448** | **0.506** |
|  | ≥2 years  (k=12, n=58) |  |  | **-0.023 [-0.315, 0.268]** | **0.874** | **-0.160** |  |  |  |  | **0.098 [-0.194, 0.390]** | **0.506** | **0.670** |  |  |
| **Rep Configuration** | Intercept (Multi reps, k=13, n=56) | **0** | **13.77%** | **0.142 [0.018, 0.266]** | **0.026** | **2.282** | **0.239** | **0.626** | **0** | **9.03%** | **0.185 [0.069, 0.301]** | **0.002** | **3.184** | **0.069** | **0.794** |
|  | Single Rep  (k=4, n=10) |  |  | **0.068 [-0.208, 0.343]** | **0.626** | **0.489** |  |  |  |  | **-0.035 [-0.303, 0.232]** | **0.794** | **-0.262** |  |  |
| **Intensity** | Intercept  (Maximal, k=10, n=42) | **0** | **10.95%** | **0.105 [-0.026, 0.237]** | **0.114** | **1.601** | **1.879** | **0.175** | **0** | **8.49%** | **0.134 [0.002, 0.265]** | **0.047** | **2.031** | **1.350** | **0.250** |
|  | Submaximal  (k=6, n=23) |  |  | **0.149 [-0.068, 0.366]** | **0.175** | **1.371** |  |  |  |  | **0.122 [-0.088, 0.333]** | **0.250** | **1.162** |  |  |
| **Intervention Modality** | Intercept  (PIMA, k=11, n=43) | **0** | **10.22%** | **0.108 [-0.020, 0.235]** | **0.096** | **1.690** | **1.896** | **0.173** | **0** | **7.92%** | **0.135 [0.008, 0.263]** | **0.038** | **2.117** | **1.364** | **0.247** |
|  | HIMA  (k=6, n=23) |  |  | **0.147 [-0.066, 0.359]** | **0.173** | **1.377** |  |  |  |  | **0.121 [-0.086, 0.328]** | **0.247** | **1.168** |  |  |
| **Sex** | Intercept  (male, k=12, n=48) | **0** | **10.3%** | **0.196 [0.077, 0.314]** | **0.002** | **3.300** | **2.533** | **0.116** | **0** | **5.18%** | **0.220 [0.110, 0.329]** | **0.0002** | **4.008** | **2.361** | **0.130** |
|  | Female  (k=3, n=14) |  |  | **-0.197 [-0.445, 0.050]** | **0.116** | **-1.592** |  |  |  |  | **-0.185 [-0.426, 0.056]** | **0.130** | **-1.537** |  |  |
| **Joint Angle** | Intercept  (≤90°, k=11, n=51) | **0** | **17.09%** | **0.107 [-0.036, 0.249]** | **0.139** | **1.501** | **3.254** | **0.076** | **0** | **14.09%** | **0.132 [-0.003, 0.268]** | **0.056** | **1.950** | **1.909** | **0.172** |
|  | 90°＜knee angle≤140°  (k=4, n=10) |  |  | **0.265 [-0.029, 0.559]** | **0.076** | **1.804** |  |  |  |  | **0.200 [-0.090, 0.490]** | **0.172** | **1.382** |  |  |
| **Volume** | Intercept  (Low, k=5, n=11) | **0** | **4.47%** | **0.205 [-0.001, 0.411]** | **0.051** | **1.988** | **3.778** | **0.028** | **0** | **2.48%** | **0.161 [-0.044, 0.367]** | **0.122** | **1.568** | **2.852** | **0.065** |
|  | Moderate Volume  (k=8, n=38) |  |  | **0.032 [-0.208, 0.271]** | **0.794** | **0.263** |  |  |  |  | **0.095 [-0.141, 0.331]** | **0.422** | **0.808** |  |  |
|  | High Volume  (k=5, n=16) |  |  | **-0.280 [-0.564, 0.004]** | **0.054** | **-1.966** |  |  |  |  | **-0.184 [-0.475, 0.107]** | **0.210** | **-1.266** |  |  |

***CI*** confidence interval**; ^*^** Moderator level significantly different from the reference level (intercept) in model without influential effect sizes**; *l^2^*** level 2**; *l^3^*** level 3**; *ES*** effect size**; *Rep*** repetition**; *HIMA*** holding isometric muscle action**; *PIMA*** pushing isometric muscle action

| **Table** Summary of moderator analysis of ISO vs CON comparison on Vertical Jump height (no intercepts) | | | | | | | | | | | | | | | |
| --- | --- | --- | --- | --- | --- | --- | --- | --- | --- | --- | --- | --- | --- | --- | --- |
| **Isometric Group vs Control Group on Vertical Jump Height (no intercepts)** | | | | | | | | | | | | | | | |
| **Moderator** | **Moderator-Level** | **Original Model** | | | | | | | **Model without Influential Studies** | | | | | | |
|  |  | ***I^2^*** | | **ES [95%CI]** | ***p*-value** | ***t*-value** | **Test of Moderator ^a^** | | ***I^2^*** | | **ES [95%CI]** | ***p*-value** | ***t*-value** | **Test of Moderator ^a^** | |
|  |  | ***l_2_*** | ***l_3_*** |  |  |  | ***F*** | ***p*** | ***l_2_*** | ***L_3_*** |  |  |  | ***F*** | ***p*** |
| **Volume^*^** | Low  (k=2, n=5) | **26.44%** | **27.87%** | **0.272 [-0.199, 0.743]** | **0.251** | **1.162** | **6.694** | **0.001** | **14.91%** | **16.43%** | **0.087 [-0.400, 0.575]** | **0.720** | **0.361** | **9.194** | **< 0.0001** |
|  | Moderate^#^  (k=7, n=29) |  |  | **0.614 [0.330, 0.898]** | **< 0.0001** | **4.345** |  |  |  |  | **0.550 [0.338, 0.761]** | **< 0.0001** | **5.243** |  |  |
|  | High  (k=5, n=14) |  |  | **0.127 [-0.236, 0.490]** | **0.485** | **0.703** |  |  |  |  | **0.075 [-0.216, 0.366]** | **0.607** | **0.519** |  |  |
| **Intensity^*^** | Maximal^#^  (k=8, n=34) | **20.94%** | **39.15%** | **0.312 [0.015, 0.608]** | **0.040** | **2.112** | **6.165** | **0.004** | **9.05%** | **32.17%** | **0.246 [0.002, 0.490]** | **0.048** | **2.033** | **7.245** | **0.002** |
|  | Submaximal^#^  (k=4, n=14) |  |  | **0.605 [0.221, 0.989]** | **0.003** | **3.167** |  |  |  |  | **0.585 [0.251, 0.919]** | **0.001** | **3.521** |  |  |
| **Contraction Duration^*^** | Short Duration  (k=2, n=5) | **18.94%** | **46.06%** | **0.360 [-0.181, 0.900]** | **0.187** | **1.337** | **2.839** | **0.048** | **9.63%** | **40.52%** | **0.199 [-0.390, 0.789]** | **0.499** | **0.681** | **2.899** | **0.045** |
|  | Moderate Duration^#^  (k=4, n=17) |  |  | **0.462 [-0.039, 0.962]** | **0.070** | **1.851** |  |  |  |  | **0.416 [0.009, 0.824]** | **0.046** | **2.057** |  |  |
|  | Long Duration^#^  (k=8, n=27) |  |  | **0.404 [0.025, 0.782]** | **0.037** | **2.144** |  |  |  |  | **0.343 [0.028, 0.659]** | **0.033** | **2.194** |  |  |
| **Rest Interval^*^** | Short Interval  (k=5, n=11) | **13.38%** | **48.27%** | **0.164 [-0.194, 0.523]** | **0.361** | **0.922** | **4.828** | **0.005** | **2.77%** | **44.67%** | **0.099 [-0.241, 0.439]** | **0.561** | **0.586** | **4.414** | **0.008** |
|  | Moderate Interval^#^  (k=10, n=29) |  |  | **0.463 [0.162, 0.765]** | **0.003** | **3.084** |  |  |  |  | **0.395 [0.134, 0.655]** | **0.004** | **3.052** |  |  |
|  | Long Interval^#^  (k=6, n=9) |  |  | **0.604 [0.210, 0.998]** | **0.003** | **3.078** |  |  |  |  | **0.455 [0.104, 0.806]** | **0.012** | **2.607** |  |  |
| **Strength Training Experience^*^** | ＜2 years  (k=2, n=3) | **18.75%** | **43.46%** | **0.338 [-0.227, 0.903]** | **0.235** | **1.201** | **4.886** | **0.011** | **8.09%** | **38.62%** | **0.175 [-0.454, 0.803]** | **0.579** | **0.559** | **4.989** | **0.011** |
|  | ≥2 years^#^  (k=10, n=46) |  |  | **0.425 [0.129, 0.720]** | **0.006** | **2.886** |  |  |  |  | **0.374 [0.132, 0.616]** | **0.003** | **3.109** |  |  |
| **Rep Configuration^*^** | Multi Reps^#^  (k=11, n=44) | **19.31%** | **42.29%** | **0.420 [0.139, 0.701]** | **0.004** | **2.999** | **5.032** | **0.010** | **8.85%** | **35.8%** | **0.371 [0.143, 0.598]** | **0.002** | **3.277** | **5.425** | **0.008** |
|  | Single Rep  (k=2, n=5) |  |  | **0.352 [-0.161, 0.865]** | **0.175** | **1.377** |  |  |  |  | **0.188 [-0.369, 0.745]** | **0.501** | **0.679** |  |  |
| **Intervention Modality^*^** | PIMA^#^  (k=9, n=35) | **20.69%** | **37.8%** | **0.311 [0.032, 0.589]** | **0.029** | **2.241** | **6.717** | **0.003** | **7.82%** | **30.88%** | **0.251 [0.023, 0.479]** | **0.032** | **2.211** | **7.992** | **0.001** |
|  | HIMA^#^  (k=4, n=14) |  |  | **0.607 [0.233, 0.980]** | **0.002** | **3.261** |  |  |  |  | **0.588 [0.263, 0.912]** | **0.001** | 3.640 |  |  |
| **Sex^*^** | Male^#^  (k=9, n=35) | **19.46%** | **47.13%** | **0.487 [0.150, 0.824]** | **0.006** | **2.908** | **4.311** | **0.019** | **10.75%** | **42.04%** | **0.363 [0.076, 0.650]** | **0.014** | **2.549** | **3.906** | **0.028** |
|  | Female  (k=3, n=11) |  |  | **0.287 [-0.316, 0.889]** | **0.343** | **0.957** |  |  |  |  | **0.375 [-0.135, 0.886]** | **0.146** | **1.481** |  |  |
| **Joint Angle^*^** | ≤90°^#^  (k=9, n=40) | **10.33%** | **57.58%** | **0.278 [-0.074, 0.630]** | **0.119** | **1.590** | **5.651** | **0.006** | **3.29%** | **49.77%** | **0.303 [0.017, 0.589]** | **0.038** | **2.136** | **5.769** | **0.006** |
|  | 90°＜Angle ≤140°^#^  (k=2, n=6) |  |  | **0.864 [0.345, 1.382]** | **0.002** | **3.352** |  |  |  |  | **0.803 [0.300, 1.305]** | **0.002** | **3.219** |  |  |
| **Overall** |  | **18.64%** | **42.01%** | **0.406 [0.152, 0.660]** | **0.0023** | **3.204** |  |  | **7.46%** | **37.58%** | **0.349 [0.128, 0.569]** | **0.003** | **3.175** |  |  |
| **Prediction Interval** |  |  |  | **[-0.560, 1.372]** |  |  |  |  |  |  | **[-0.365, 1.062]** |  |  |  |  |

***CI* c**onfidence interval**; ^*^** Test of moderator reached significance in model without influential effect sizes**; ^#^** Moderator levels reached significance in model without influential effect sizes**; *l ^2^*** level 2**; *l ^3^*** level 3**; *ES*** effect size**; *Rep*** repetition**; *HIMA*** holding isometric muscle action**; *PIMA*** pushing isometric muscle action

| **Table** Summary of moderator analysis of ISO vs CON comparison on Vertical Jump height (with intercepts) | | | | | | | | | | | | | | | |
| --- | --- | --- | --- | --- | --- | --- | --- | --- | --- | --- | --- | --- | --- | --- | --- |
| **Isometric Group vs Control Group on Vertical Jump Height (with intercepts)** | | | | | | | | | | | | | | | |
| **Moderator** | **Moderator-Level** | **Original Model** | | | | | | | **Model without Influential Studies** | | | | | | |
|  |  | ***I^2^*** | | **ES [95%CI]** | ***p*-value** | ***t*-value** | **Test of Moderator ^a^** | | ***I^2^*** | | **ES [95%CI]** | ***p*-value** | ***t*-value** | **Test of Moderator ^a^** | |
|  |  | ***l_2_*** | ***l_3_*** |  |  |  | ***F*** | ***p*** | ***l_2_*** | ***L_3_*** |  |  |  | ***F*** | ***p*** |
| **Contraction Duration** | Intercept  (Short, k=2, n=5) | **18.94%** | **46.06%** | **0.360 [-0.181, 0.900]** | **0.187** | **1.337** | **0.039** | **0.961** | **9.63%** | **40.52%** | **0.199 [-0.390, 0.789]** | **0.499** | **0.681** | 0.188 | **0.829** |
|  | Moderate  (k=4, n=17) |  |  | **0.102 [-0.631, 0.835]** | **0.781** | **0.279** |  |  |  |  | **0.217 [-0.497, 0.931]** | **0.544** | **0.612** |  |  |
|  | Long  (k=8, n=27) |  |  | **0.044 [-0.562, 0.649]** | **0.885** | **0.146** |  |  |  |  | **0.144 [-0.478, 0.766]** | **0.643** | **0.467** |  |  |
| **Rest Interval** | Intercept  (Short, k=5, n=11) | **13.38%** | **48.27%** | **0.164 [-0.194, 0.523]** | **0.361** | **0.922** | **2.634** | **0.082** | **2.77%** | **44.67%** | **0.099 [-0.241, 0.439]** | **0.561** | **0.586** | **2.292** | **0.113** |
|  | Moderate  (k=10, n=29) |  |  | **0.299 [-0.062, 0.659]** | **0.102** | **1.665** |  |  |  |  | **0.296 [-0.035, 0.627]** | **0.079** | **1.798** |  |  |
|  | Long  (k=6, n=9) |  |  | **0.439 [0.038, 0.840]** | **0.032** | **2.201** |  |  |  |  | **0.356 [-0.014, 0.726]** | **0.059** | **1.935** |  |  |
| **Strength Training Experience** | Intercept  (＜2 years, k=2, n=3) | **18.75%** | **43.46%** | **0.338 [-0.227, 0.903]** | **0.235** | **1.201** | **0.075** | **0.786** | **8.09%** | **38.62%** | **0.175 [-0.454, 0.803]** | **0.579** | **0.559** | **0.355** | **0.554** |
|  | ≥2 years  (k=10, n=46) |  |  | **0.087 [-0.551, 0.724]** | **0.786** | **0.273** |  |  |  |  | **0.200 [-0.474, 0.873]** | **0.554** | **0.596** |  |  |
| **Rep Configuration** | Intercept (Multi reps, k=11, n=44) | **19.31%** | **42.29%** | **0.420 [0.139, 0.701** | **0.004** | **2.999** | **0.061** | **0.807** | **8.85%** | **35.8%** | **0.371 [0.143, 0.598]** | **0.002** | **3.277** | **0.403** | **0.529** |
|  | Single Rep  (k=2, n=5) |  |  | **-0.068 [-0.624, 0.487]** | **0.807** | **-0.246** |  |  |  |  | **-0.183 [-0.761, 0.396]** | **0.529** | **-0.635** |  |  |
| **Intensity** | Intercept  (Maximal, k=8, n=34) | **20.94%** | **39.15%** | **0.312 [0.015, 0.608]** | **0.040** | **2.112** | **1.826** | **0.183** | **9.05%** | **32.17%** | **0.246 [0.002, 0.490]** | **0.048** | **2.033** | **3.248** | **0.078** |
|  | Submaximal  (k=4, n=14) |  |  | **0.294 [-0.143, 0.730]** | **0.183** | **1.351** |  |  |  |  | **0.339 [-0.040, 0.717]** | **0.078** | **1.802** |  |  |
| **I****ntervention Modality** | Intercept  (PIMA, k=9, n=35) | **20.69%** | **37.8%** | **0.311 [0.032, 0.589]** | **0.029** | **2.241** | **1.970** | **0.167** | **7.82%** | **30.88%** | **0.251 [0.023, 0.479]** | **0.032** | **2.211** | **3.430** | **0.070** |
|  | HIMA  (k=4, n=14) |  |  | **0.296 [-0.127, 0.719]** | **0.167** | **1.404** |  |  |  |  | **0.337 [-0.029, 0.703]** | **0.070** | **1.852** |  |  |
| **Sex** | Intercept  (male, k=9, n=35) | **19.46%** | **47.13%** | **0.487 [0.150, 0.824]** | **0.006** | **2.908** | **0.408** | **0.526** | **10.75%** | **42.04%** | **0.363 [0.076, 0.650]** | **0.014** | **2.549** | **0.002** | **0.965** |
|  | Female  (k=3, n=11) |  |  | **-0.200 [-0.831, 0.430]** | **0.526** | **-0.639** |  |  |  |  | **0.012 [-0.539, 0.563]** | **0.965** | **0.045** |  |  |
| **Joint Angle** | Intercept  (≤90°, k=9, n=40) | **10.33%** | **57.58%** | **0.278 [-0.074, 0.630]** | **0.119** | **1.590** | **5.660** | **0.022** | **3.29%** | **49.77%** | **0.303 [0.017, 0.589]** | **0.038** | **2.136** | **4.325** | **0.043** |
|  | 90°＜knee angle≤140°^*^  (k=2, n=6) |  |  | **0.586 [0.091, 1.081** | **0.022** | **2.379** |  |  |  |  | **0.500 [0.015, 0.984]** | **0.043** | **2.080** |  |  |
| **Volume** | Intercept  (Low, k=2, n=5) | **26.44%** | **27.87%** | **0.272 [-0.199, 0.743]** | **0.251** | **1.162** | **2.849** | **0.068** | **14.91%** | **16.43%** | **0.087 [-0.400, 0.575]** | **0.720** | **0.361** | **4.499** | **0.017** |
|  | Moderate  (k=7, n=29) |  |  | **0.341 [-0.204, 0.887]** | **0.214** | **1.259** |  |  |  |  | **0.462 [-0.068, 0.992]** | **0.086** | **1.757** |  |  |
|  | High  (k=5, n=14) |  |  | **-0.145 [-0.708, 0.417]** | **0.606** | **-0.520** |  |  |  |  | **-0.012 [-0.562, 0.537]** | **0.964** | **-0.045** |  |  |

***CI*** confidence interval**; ^*^** Moderator level significantly different from the reference level (intercept) in model without influential effect sizes**; *l^2^*** level 2**; *l^3^*** level 3**; *ES*** effect size**; *Rep*** repetition**; *HIMA*** holding isometric muscle action**; *PIMA*** pushing isometric muscle action
