# Supplementary material for: From Populations to Programming Variables: Post‐Activation Performance Enhancement Following Isometric Contractions on Muscular Strength and Power—A Systematic Review and Meta‐Analysis
Source: Eur J Sport Sci. 2026 May 19;26(6):e70183. doi: 10.1002/ejsc.70183 (PMC13580768; doi:10.1002/ejsc.70183)
Supplement: Supplementary file 10 [file EJSC-26-e70183-s010.docx]

**Supplementary File Legends**

Supplementary File 1. Search string.

Supplementary File 2. Data extraction, conversion, and imputation procedures.

Supplementary File 3. Coding of the study.

Supplementary File 4. Risk of bias assessment.

Supplementary File 5. Study quality evaluation.

Supplementary File 6. Tables of moderator analysis results.

Supplementary File 7. Meta-regression model details.

Supplementary File 8. GRADE assessment results.

Supplementary File 9. Additional methods and results for the meta-analyses.
